# Potentiation of ΔF508- and G551D-CFTR-Mediated Cl^-^ Current by Novel Hydroxypyrazolines

**DOI:** 10.1371/journal.pone.0149131

**Published:** 2016-02-10

**Authors:** Jinhong Park, Poonam Khloya, Yohan Seo, Satish Kumar, Ho K. Lee, Dong-Kyu Jeon, Sungwoo Jo, Pawan K. Sharma, Wan Namkung

**Affiliations:** 1 College of Pharmacy, Yonsei Institute of Pharmaceutical Sciences, Yonsei University, Incheon 406–840, Korea; 2 Department of Integrated OMICS for Biomedical Science, WCU Program of Graduate School, Yonsei University, Seoul 120–749, Korea; 3 Department of Chemistry, Kurukshetra University, Kurukshetra, Haryana 136119, India; University Medical Center Utrecht, NETHERLANDS

## Abstract

The most common mutation of CFTR, affecting approximately 90% of CF patients, is a deletion of phenylalanine at position 508 (F508del, ΔF508). Misfolding of ΔF508-CFTR impairs both its trafficking to the plasma membrane and its chloride channel activity. To identify small molecules that can restore channel activity of ΔF508-CFTR, we synthesized and evaluated eighteen novel hydroxypyrazoline analogues as CFTR potentiators. To elucidate potentiation activities of hydroxypyrazolines for ΔF508-CFTR, CFTR activity was measured using a halide-sensitive YFP assay, Ussing chamber assay and patch-clamp technique. Compounds **7p**, **7q** and **7r** exhibited excellent potentiation with EC_50_ value <10 μM. Among the compounds, **7q** (a novel CFTR potentiator, CP7q) showed the highest potentiation activity with EC_50_ values of 0.88 ± 0.11 and 4.45 ± 0.31 μM for wild-type and ΔF508-CFTR, respectively. In addition, CP7q significantly potentiated chloride conductance of G551D-CFTR, a CFTR gating mutant; its maximal potentiation activity was 1.9 fold higher than the well-known CFTR potentiator genistein. Combination treatment with CP7q and VX-809, a corrector of ΔF508-CFTR, significantly enhanced functional rescue of ΔF508-CFTR compared with VX-809 alone. CP7q did not alter the cytosolic cAMP level and showed no cytotoxicity at the concentration showing maximum efficacy. The hydroxypyrazolines may be potential development candidates for drug therapy of cystic fibrosis.

## Introduction

Cystic fibrosis (CF) is caused by loss of function mutations in the gene encoding CFTR, a cAMP-dependent chloride channel expressed primarily in the airways, pancreas, intestine and other organs [1–4]. The most common mutation responsible for CF is deletion of phenylalanine at residue 508 (F508del, ΔF508) and this mutation causes protein misfolding that leads to defects in trafficking to the plasma membrane. Even though ΔF508-CFTR was properly localized to the plasma membrane by low temperature correction or small-molecule correctors, the ΔF508-CFTR has defects in channel gating [5], plasma membrane stability [6] and thermal stability [7, 8]. G551D-CFTR mutation is found in about 4~5% of CF patients, and this mutation produces defects primarily in CFTR channel gating, but the expression, processing and localization of this mutant protein remains normal [9].

To treat people with one or two copies of the F508del mutation, various F508del-CFTR correctors have been identified including Corr-4a, VX-809, VX-325, KM11060, ibruprofen and trimethylangelicin [10–14]. VX-809 is a promising investigational corrector of ΔF508-CFTR that restores plasma membrane expression of ΔF508-CFTR and chloride channel function to approximately 14% of non-CF human bronchial epithelial cells [11]. In clinical trials, however, VX-809 treatment was able to improve functional expression of ΔF508-CFTR in the sweat gland but failed to show robust improvement in lung function of patients with CF who were homozygous for the F508del-CFTR mutation [15]. To overcome the limitations of first-generation correctors such as VX-809, a recent study on identification of small-molecule correctors showing synergy with VX-809 provided a different types of correctors that can increase efficacy when used in combination [16–18].

The gating defects of corrected ΔF508-CFTR and other gating mutants, such as G551D, have led to the development of small-molecule potentiators that can enhance CFTR Cl^-^ channel activity. Several small-molecule compounds have been found to have potentiation activity for ΔF508- and G551D-CFTR: VX-770, genistein, apigenin, phenylglycine-01 (PG-01) and VX-532 to name a few [19–23]. Kalydeco (ivacaftor, VX-770) is the first approved potentiator for the treatment of CF patients with the G551D-CFTR mutation [24] or some other rare gating mutations including G178R, S549N, S549R, G551S, G1244E, S1251N, S1255P and G1349D mutation [25]. VX-770 treatment of patients with G551D and other gating mutations demonstrated noticeable clinical improvements, including increase in the forced expiratory volume in 1 s (FEV1), decrease in pulmonary exacerbations, and weight gain compared to placebo group [26, 27]. The studies on VX-770 clearly show that the corrector is a good therapeutic agent for people with CF who have gating mutations. Vertex Pharmaceuticals Inc. reported results of Phase 3 clinical trials of ivacaftor (VX-770) in combination with lumacaftor (VX-809) for CF patients who are homozygous for F508del-CFTR. The combination therapy revealed significant improvements in lung function and other key measures of the disease. In addition, U.S. Food and Drug Administration (FDA) approved the combination therapy, Orkambi (lumacaftor/ivacaftor), to treat the underlying cause of CF in people aged 12 or older who are homozygous for F508del-CFTR mutation on July 2, 2015 (www.vrtx.com). However, recent study showed that prolonged incubation with most of known potentiators including VX-770 and genistein reduced the correction efficacy of VX-809 and VX-661, an investigational corrector [28, 29]. This result strongly suggests that the identification and optimization of potentiators are still needed to improve the clinical benefit of combination therapy of corrector and potentiator in CF.

Careful study of literature revealed that diverse groups of compounds such as bithiazoles, pyrazolylthiazoles, benzoquinoliziums, xanthines, benzimidazoles and flavonoids possessing central heterocyclic rings have been found to exhibit CFTR corrector and/or potentiator activity [30–34]. Common features of most of these compounds happen to be the presence of a heterocyclic moiety as well as the presence of a hydroxyl group or other groups capable of hydrogen bonding. In order to broaden the scope of our ongoing research program in the field of heterocyclic compounds of potential medicinal interest [35–38], we designed and synthesized a series of eighteen novel hydroxypyrazoline analogues bearing benzenesulfonamide and at least one free hydroxyl group for evaluation as candidates for potentiation of CFTR.

To elucidate the effect of the hydroxypyrazolines on WT, ΔF508- and G551D-CFTR chloride channel activity and their potential usefulness for drug development, we carried out electrophysiological studies in cell lines overexpressing CFTR and in primary airway epithelium. CP7q exhibited the highest activity and strongly potentiated ΔF508- and G551D-CFTR. The hydroxypyrazolines may provide new structural scaffolds for CF therapeutics development.

## Methods

### Materials and solutions

Forskolin, genistein and other chemicals, unless otherwise indicated, were purchased from Sigma. CFTR_inh_-172 was synthesized as described. The HCO_3_^—^buffered solution contained (in mM): 120 NaCl, 5 KCl, 1 MgCl_2_, 1 CaCl_2_, 10 D-glucose, 5 HEPES, and 25 NaHCO_3_ (pH 7.4). In the half-Cl^-^ solution, 65 mM NaCl in the HCO_3_^—^buffered solution was replaced by Na gluconate.

### Cell culture

Fisher rat thyroid (FRT) cells expressing human wild type-, ΔF508- and G551D-CFTR with a halide sensor YFP-H148Q/I152L were generously provided by Dr. Alan Verkman (University of California, San Francisco) and grown in F-12 Modified Coon´s medium supplemented with 10% FBS, 2 mM glutamine, 100 units/ml penicillin and 100 μg/ml streptomycin [30]. A549 cells were obtained from Korean Cell Line Bank (Seoul, Korea) and the cells were stably transfected with human ΔF508-CFTR and the halide sensor YFP-H148Q/I152L/F46L, and maintained in RPMI-1640 medium containing 10% fetal bovine serum (FBS), 100 units/ml penicillin and 100 μg/ml streptomycin. HT-29 cells were maintained in DMEM medium containing 10% fetal bovine serum (FBS), 100 units/ml penicillin and 100 μg/ml streptomycin. Primary cultures of human nasal epithelial cells were generously provided by Dr. Jaeyoung Choi. Passage-2 human nasal epithelial cells were plated at a density of 1 x10^5^ per cm^2^ onto 12-mm diameter, 0.4-μm pore polycarbonate cell culture inserts (Snapwell; Corning, Lowell, MA). The cells were maintained in 1:1 mixture of bronchial epithelial growth medium and Dulbecco’s modified Eagle’s medium containing 10% fetal bovine serum and all supplements [39]. The cells were grown at an air-liquid interface and medium was changed every 2–3 days. Cultures were used 21 days after plating at which time transepithelial resistance was 300–800 Ohm/cm^2^.

### Microplate Reader Assay of CFTR potentiation

FRT cells expressing wild type- or ΔF508-CFTR with the halide sensor YFP-H148Q/I152L were plated in 96-well black-walled microplates (Corning Inc., Corning, NY) at a density of 2 x10^4^cells per well. FRT-WT-CFTR-YFP cells were incubated for 48 h at 37°C, and FRT-ΔF508-CFTR-YFP cells were incubated for 24 h at 27°C after 24 h incubation at 37°C to rescue ΔF508-CFTR localization. Assays were done using FLUO star Omega microplate reader (BMG Labtech, Ortenberg, Germany) and MARS Data Analysis Software (BMG Labtech). Each well of a 96-well plate was washed 3 times in PBS (200 μL/wash). 100 μL PBS was added to each well. Forskolin (0.1 and 10 μM for WT- and ΔF508-CFTR, respectively) and test compounds (1 μL) were added to each well. After 10 min, 96-well plates were transferred to the microplate reader preheated to 37°C for fluorescence assay. Each well was assayed individually for CFTR-mediated I^-^ influx by recording fluorescence continuously (200 ms per point) for 2 s (baseline), then 100 μL of 140 mM I^-^ solution was added at 2 s and then YFP fluorescence was recorded for 14 s. Initial iodide influx rate was determined from the initial slope of fluorescence decrease, by nonlinear regression, following infusion of iodide.

### Short-circuit Current

Snapwell inserts containing CFTR-expressing FRT and primary culture of human nasal epithelial cells were mounted in Ussing chambers (Physiologic Instruments, San Diego, CA). Forskolin, genistein, CP7q and CFTR_inh_-172 were added to the apical and basolateral bath solution. For FRT cells, the apical bath was filled with a half-Cl^-^ solution and the basolateral bath was filled with HCO_3_^—^buffered solution, and the basolateral membrane was permeabilized with 250 μg/mL amphotericin B. For primary cultures of human nasal epithelial cells, symmetrical HCO_3_^—^buffered solutions were used and ENaC was inhibited by pre-treatment with amiloride (100 μM). All cells were bathed for a 20 min stabilization period and aerated with 95% O_2_ / 5% CO_2_ at 37°C. Apical membrane current and short-circuit current were measured with an EVC4000 Multi-Channel V/I Clamp (World Precision Instruments, Sarasota, FL) and recorded using PowerLab 4/35 (AD Instruments, Castle Hill, Australia). Data were collected and analyzed with ADInstruments acquisition software Labchart Pro 7 software. The sampling rate was 4 Hz.

### Patch-Clamp

Whole-cell patch-clamp recordings were performed on WT- or ΔF508-CFTR-expressing FRT cells. The bath solution contained (in mM): 140 NMDG-Cl, 1 CaCl_2_, 1 MgCl_2_, 10 glucose and 10 HEPES (pH 7.4). The pipette solution contained (in mM): 130 CsCl, 0.5 EGTA, 1 MgCl_2_, 1 Tris-ATP, and 10 HEPES (pH 7.2). Pipettes were pulled from borosilicate glass and had resistances of 3–5 MΩ after fire polishing. Seal resistances were between 3 and 10 GΩ. After establishing the whole-cell configuration, CFTR was activated by forskolin and/or CP7q. Whole-cell currents were elicited by applying hyperpolarizing and depolarizing voltage pulses from a holding potential of 0 mV to potentials between -80 mV and +80 mV in steps of 20 mV. Recordings were made at room temperature using an Axopatch-200B (Axon Instruments). Currents were digitized and analyzed using a Digidata 1440A converter (Axon Instruments), and pCLAMP 10.2 software (Molecular Devices, Sunnyvale, CA). Currents were low-pass filtered at 1 kHz and sampled at 5 kHz

### Cyclic AMP Assay

FRT cells grown on 12 well culture plates were washed 3 times with PBS at 37°C and then incubated in PBS at 37°C containing 100 μM IBMX for 5 min in the absence or presence of forskolin or CP7q. After 10 min incubation, the cells were washed with cold PBS and cytosolic cAMP was measured using a cAMP immunoassay kit (Parameter TM cAMP immunoassay kit, R&D Systems) according to the manufacturer's protocol.

### Cell Proliferation Assays

HT-29 human colon adenocarcinoma cells were plated at a density of 1 x10^4^ per cm^2^on 96-well microplates. After 24 h incubation, cells were treated with hydroxypyrazolines and then incubated for 1 day. An equal amount of DMSO was added to the all control. The culture medium and the compounds were changed every 12 h. For MTS assay, the cells were reincubated with MTS for 1 hour. The soluble formazan produced by cellular reduction of MTS was quantified by measuring the absorbance at 490 nm with Infinite M200 (Tecan, Grödig, Austria) microplate reader. MTS assay was done using CellTiter 96^®^ AQueous One Solution Cell Proliferation Assay kit (Promega, Madison, WI, USA).

### Immunoblot

A549 cells stably expressing ΔF508-CFTR were lysed with cell lysis buffer (50 mM Tris-HCl, pH 7.4, 1% Nonidet P-40, 0.25% sodium deoxycholate, 150 mM NaCl, 1 mM EDTA, 1 mM Na_3_VO_4_, and protease inhibitor mixture). Whole cell lysates were centrifuged at 15,000 g for 10 min at 4°C to remove the cell debris, and equal amounts (80 μg protein/lane) of supernatant protein were separated by 4–12% Tris-glycine precast gel (KOMA BIOTECH, Seoul, Korea) and then transferred onto PVDF membrane (Millipore, Billerica, MA). Membrane was blocked with 5% non-fat skim milk in PBS including 0.05% Tween 20 for 1 hour at room temperature. This membrane was then incubated overnight with primary CFTR antibody (M3A7, Millipore, Billerica, MA). After washing with 0.05% Tween 20 in PBS (PBST), the blot was further incubated for 45 min at room temperature with an anti-mouse secondary antibody (Cell Signaling). The membrane was then washed three times with PBST for 5 minutes and then visualized using the ECL Plus western blotting detection system (GE Healthcare Amersham; Piscataway, NJ). The immunoblot results were analyzed quantitatively by ImageJ Software.

### Statistical analysis

The results of multiple experiments are expressed as means ± SEM of *n* observations. Statistical analysis was performed with Student’s t-test or by analysis of variance as appropriate. Differences were considered statistically significant when *P* < 0.05.

## Results

### Potentiation of CFTR by Novel Hydroxypyrazolines

**Fig 1:**
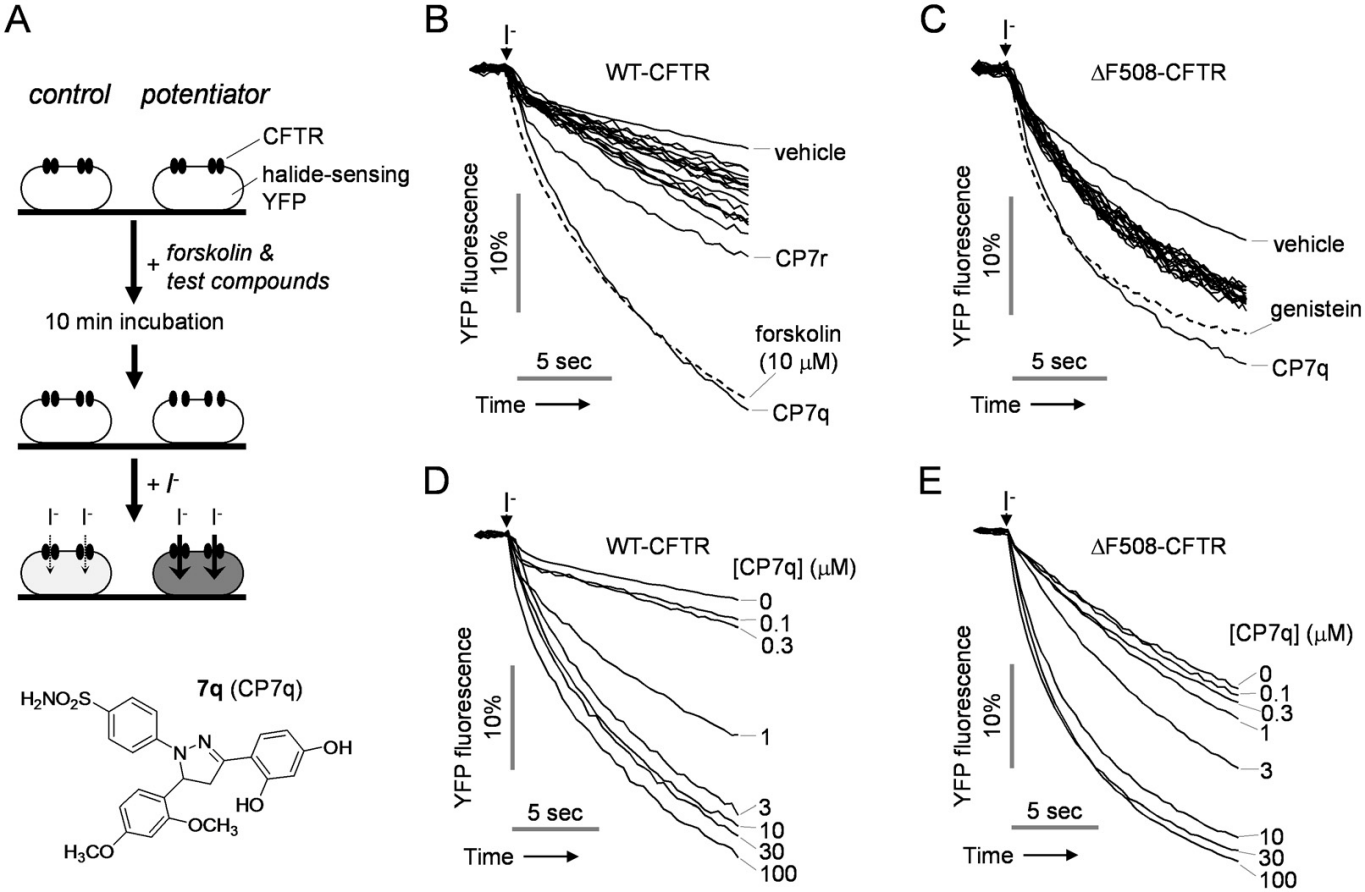
Identification of novel potentiators of human CFTR. (A) Assay protocol. FRT cells stably expressing the halide-sensitive cytoplasmic fluorescent sensor YFP-148Q/I152L and CFTR were incubated with forskolin and test compounds. The YFP fluorescence was measured in response to addition of iodide (top panel). Chemical structure of CP7q (bottom panel). (B) In WT-CFTR expressing FRT cells, the YFP fluorescence measured in single wells of 96-well plates, showing vehicle control, 10 μM forskolin and 3 μM hydroxypyrazolines in the presence of 0.1 μM forskolin. (C) In FRT cells expressing ΔF508-CFTR that has been rescued by low temperature, the YFP fluorescence measured in single wells of 96-well plates, showing vehicle control, 50 μM genistein and 3 μM hydroxypyrazolines in the presence of 10 μM forskolin. (D) Representative traces showing iodide influx via WT-CFTR in the presence of the indicated concentrations of CP7q and 0.1 μM forskolin. (E) Representative traces showing iodide influx via low temperature-rescued ΔF508-CFTR in the presence of the indicated concentrations of CP7q and 10 μM forskolin.

To investigate whether novel hydroxypyrazolines potentiate CFTR Cl^-^ channel activity, FRT cells were stably transfected with iodide-sensitive YFP and CFTR, and potentiation activity was tested using a cell-based fluorescence assay (Fig 1A). Iodide influx through CFTR was measured by the addition of extracellular iodide after pre-incubation with the hydroxypyrazolines and forskolin, a cAMP agonist. Among the hydroxypyrazolines, 4-[3-(2,4-dihydroxyphenyl)-5-(2,4-dimethoxyphenyl)-4,5-dihydro-1H-pyrazol-1-yl] benzenesulfonamide (CP7q) most strongly potentiated WT- and ΔF508-CFTR channel activity when CFTR was activated by 0.1 and 10 μM forskolin in WT- andΔF508-CFTR expressing FRT cells, respectively (Fig 1B–1E). EC_50_ values of the hydroxypyrazolines for WT- and ΔF508-CFTR calculated from microplate reader assay are given in Table 1.

**Table 1:** CFTR potentiation by hydroxy pyrazoline compounds. Structure-activity relationship of CFTR potentiator is shown. EC_50_ values were determined by microplate reader assay.

Comp No.	R^1^	R^2^	R^3^	R^4^	R^5^	R^6^	EC_50_ (μM) WT- CFTR	EC_50_ (μM) ΔF508-CFTR
**7a**	H	H	H	OCH_3_	OCH_3_	H	18.5	45.6
**7b**	H	H	H	OCH_3_	H	OCH_3_	32.4	68.8
**7c**	H	H	H	H	OCH_3_	OCH_3_	10.3	30.1
**7d**	H	H	OCH_3_	OCH_3_	OCH_3_	H	56.5	inactive
**7e**	H	H	OCH_3_	OCH_3_	H	OCH_3_	63.3	inactive
**7f**	H	H	OCH_3_	H	OCH_3_	OCH_3_	50.6	inactive
**7g**	H	CH_3_	H	OCH_3_	OCH_3_	H	inactive	inactive
**7h**	H	CH_3_	H	OCH_3_	H	OCH_3_	10.9	inactive
**7i**	H	CH_3_	H	H	OCH_3_	OCH_3_	38.6	83
**7j**	H	Cl	H	OCH_3_	OCH_3_	H	92.5	inactive
**7k**	H	Cl	H	OCH_3_	H	OCH_3_	13.1	inactive
**7l**	H	Cl	H	H	OCH_3_	OCH_3_	41.2	inactive
**7m**	H	H	OH	OCH_3_	OCH_3_	H	8.7	42.2
**7n**	H	H	OH	OCH_3_	H	OCH_3_	27	38.5
**7o**	H	H	OH	H	OCH_3_	OCH_3_	24.3	inactive
**7p**	OH	H	H	OCH_3_	OCH_3_	H	12.7	8.6
**7q**	OH	H	H	OCH_3_	H	OCH_3_	0.9	4.8
**7r**	OH	H	H	H	OCH_3_	OCH_3_	2.4	8.9

### Synthesis of Hydroxypyrazolines

**Fig 2:**
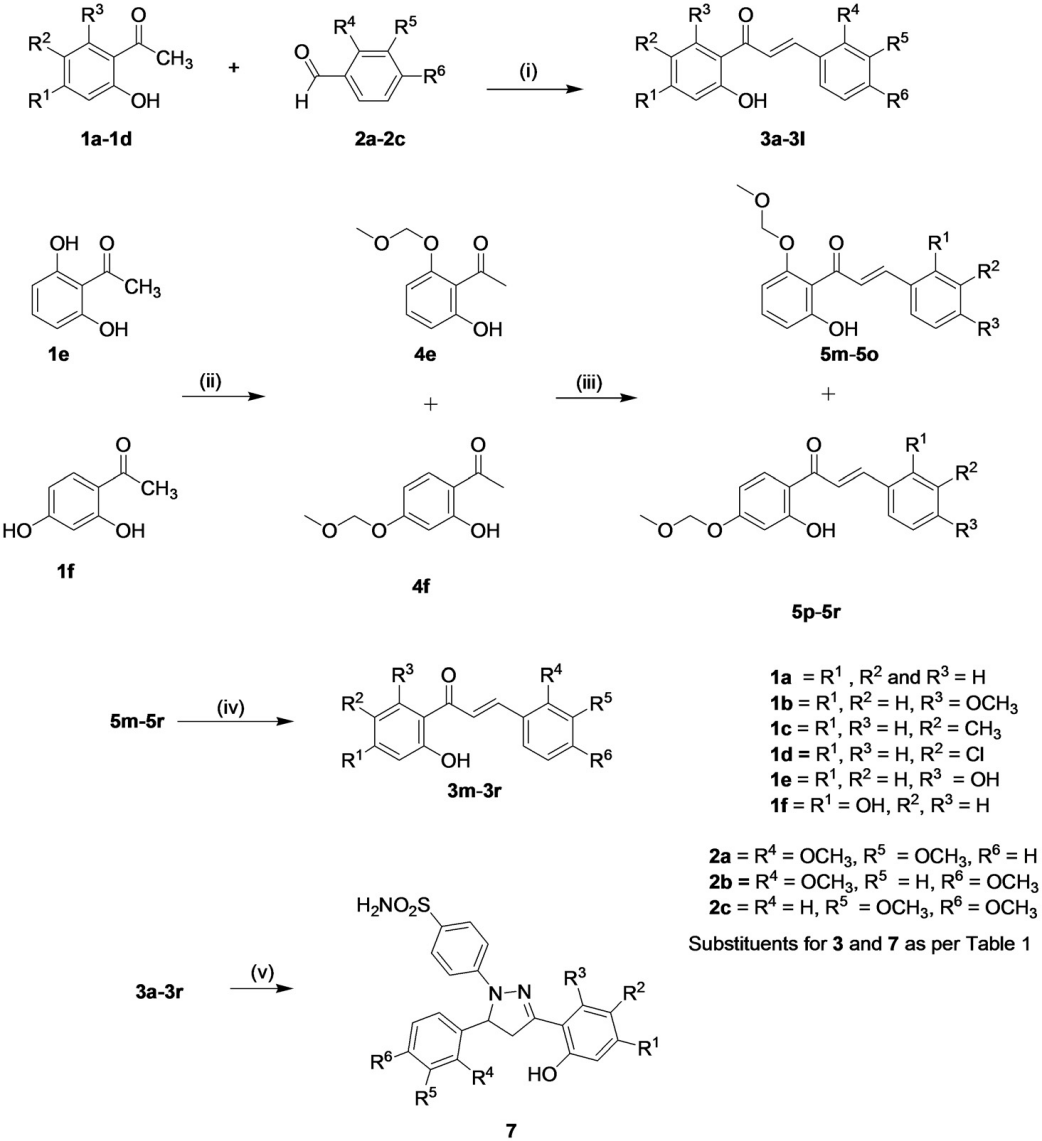
Synthesis of the Hydroxy Pyrazolines as CFTR potentiators. (i) KOH, EtOH, stir; (ii) Chloro(methoxy)methane (MOMCl), K_2_CO_3_, Acetone, reflux; (iii) substituted benzaldehydes 2a-2c, KOH, EtOH, stir; (iv) 3N HCl, reflux; (v) 4-hydrazinobenzenesulfonamide hydrochloride (see Materials and Methods).

The target hydroxypyrazolines (7a-7r) were synthesized by following synthetic route outlined in Fig 2. The synthesis of hydroxypyrazolines rest on the intermediate hydroxychalcones 3a-3r which were synthesized by base catalyzed Claisen-Schmidt condensation reaction of the appropriate acetophenones 1 with substituted benzaldehydes 2 [40]. The target hydroxypyrazolines 7a-7r were obtained by condensation of appropriate chalcones 3a-3r with 4-hydrazinobenzenesulfonamide hydrochloride [36] in refluxing acidic ethanol. 4-Hydrazinobenzenesulfonamide hydrochloride in turn was prepared *via* diazotization of sulfanilamide followed by reduction of the corresponding diazonium salt with stannous chloride [41].

It is pertinent to mention here that six acetophenones (1a-1f) were selected for the present study possessing at least one hydroxy group which was present at position-2. In case of dihydroxy acetophenones 1e and 1f, second hydroxy group was present at position-6 and position-4 respectively. This additional hydroxyl group in 1e and 1f had to be protected using chloro(methoxy)methane in refluxing acetone under basic conditions [42]. After the successful synthesis of protected chalcones 5m-5r, the deprotection of hydroxy group was achieved by refluxing in 3N HCl solution to afford chalcones 3m-3r as shown in Fig 2.

The structures of all the synthesized chalcones 5m-5r, 3a-3r and hydroxypyrazolines 7a-7r was confirmed by their spectral (IR, ^1^H NMR and ^13^C NMR) data. In the ^1^H NMR spectra of compounds 5m-5r, the presence of methylene protons flanked by two oxygen atoms corresponding to methoxymethoxy (MOM) group could be ascertained from a singlet resonating at ~ δ 5.30, which was further supported from ^13^C NMR spectra exhibiting signal at ~ δ 95.3. The IR spectra of chalcones 3a-3r exhibited the characteristic absorption band for C = O stretching at ~1628 cm^-1^. All the synthesized chalcones in the present study were found to be geometrically pure with *trans*-configuration as indicated by the coupling constant (*J*_Hα-Hβ_ = 15.6–15.3 Hz) in their ^1^H NMR spectra. In general, ^1^H NMR spectra of pyrazolines 7a-7r showed characteristic ABX pattern of three protons of pyrazoline including two at C4 and one at C5. C_5_-H of pyrazoline resonated at ~ δ 5.70 as a doublet of doublet with coupling constants of 12.6 Hz and 5.6 Hz. The cis C_4_-H appeared as a doublet of doublet at ~ δ 4.11 with coupling constants 18.0 Hz and 12.6 Hz. The trans C_4_-H also appeared as a doublet of doublet at ~ δ 3.32 with coupling constants 18.0 Hz and 5.6 Hz. The structure of 7a-7r was further supported by their ^13^ C NMR by the presence of signals ~ δ 60.7 and ~ δ 43.5 due to C_5_- and C_4_-pyrazoline carbon atoms further confirmed the pyrazoline structure. A singlet exchangeable in D_2_O, present at ~ δ 7.04 could be assigned to the SO_2_NH_2_ group present at 4-position of phenyl ring attached to *N*-1 of pyrazoline ring. The experimental protocols of all synthesized compounds are presented in S1 file.

### Structure-Activity Relationship Analysis of Hydroxypyrazolines

In the present study a careful look at CFTR potentiation data and substitution pattern of newly synthesized hydroxypyrazolines 7 revealed that three compounds namely, 7p, 7q, 7r having 2,4-dihydroxyphenyl moiety attached at C3 of pyrazoline ring were found to be excellent ΔF508-CFTR potentiators with EC_50_ value in the range 4.8–8.9 M while compounds 7q and 7r with EC_50_ values 0.9 M and 2.4 M respectively were excellent WT-CFTR potentiators. Among the eighteen compounds 7a-7r, compound 7q having hydroxy and methoxy substituents in the phenyl rings (Table 2) attached at C3 and C5 of pyrazoline ring exhibited best potentiation against both ΔF508-CFTR and WT-CFTR. Other fifteen compounds 7a-7o having hydroxy group at position-2 in the phenyl ring at C3 and other substituents at various positions exhibit varying degree of potentiation without following any definite trend. Presence of free hydroxy group at position-4 of phenyl ring at C3 and methoxy group at position-4 of phenyl ring at C5 of pyrazoline nucleus exhibited the best results. A more detailed and elaborate study is further required to establish a precise structure activity relationship.

**Table 2:** Substitution pattern of hydroxypyrazolines (7a-7r) and intermediate hydroxychalcones (3a-3r).

Comp No.	R^1^	R^2^	R^3^	R^4^	R^5^	R^6^
**3a**, **7a**	H	H	H	OCH_3_	OCH_3_	H
**3b**, **7b**	H	H	H	OCH_3_	H	OCH_3_
**3c**, **7c**	H	H	H	H	OCH_3_	OCH_3_
**3d**, **7d**	H	H	OCH_3_	OCH_3_	OCH_3_	H
**3e**, **7e**	H	H	OCH_3_	OCH_3_	H	OCH_3_
**3f**, **7f**	H	H	OCH_3_	H	OCH_3_	OCH_3_
**3g**, **7g**	H	CH_3_	H	OCH_3_	OCH_3_	H
**3h**, **7h**	H	CH_3_	H	OCH_3_	H	OCH_3_
**3i**, **7i**	H	CH_3_	H	H	OCH_3_	OCH_3_
**3j**, **7j**	H	Cl	H	OCH_3_	OCH_3_	H
**3k**, **7k**	H	Cl	H	OCH_3_	H	OCH_3_
**3l**, **7l**	H	Cl	H	H	OCH_3_	OCH_3_
**3m**, 7**m**	H	H	OH	OCH_3_	OCH_3_	H
**3n**, **7n**	H	H	OH	OCH_3_	H	OCH_3_
**3o**, **7o**	H	H	OH	H	OCH_3_	OCH_3_
**3p**, **7p**	OH	H	H	OCH_3_	OCH_3_	H
**3q**, **7q**	OH	H	H	OCH_3_	H	OCH_3_
**3r**, **7r**	OH	H	H	H	OCH_3_	OCH_3_

### CP7q Potentiates WT-CFTR Channel Activity

**Fig 3:**
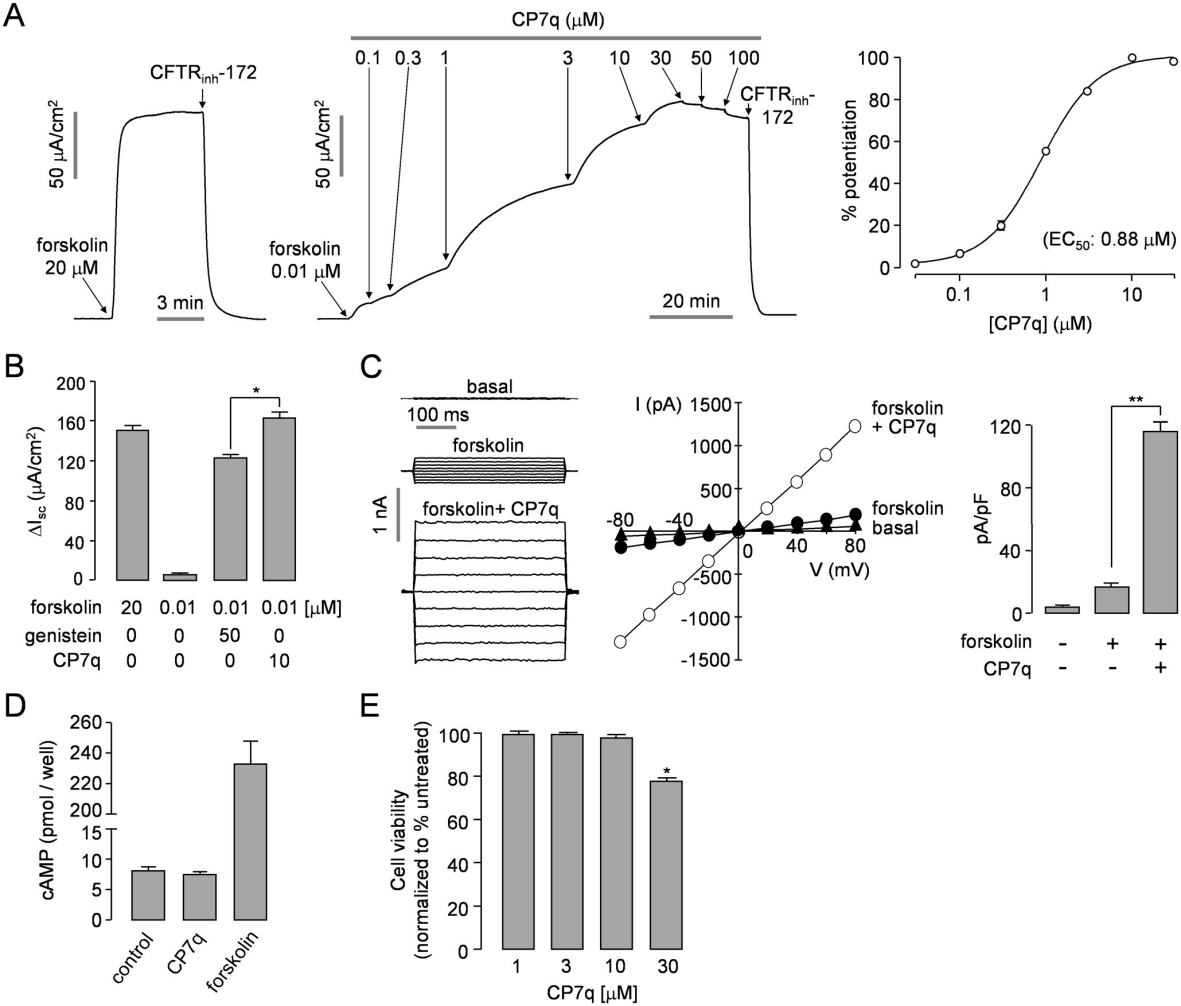
Characterization of CP7q, a small-molecule potentiator of CFTR. (A) Apical membrane currents measured in FRT cells expressing WT-CFTR. CFTR was potentiated by indicated concentrations of forskolin and CP7q (left and middle panel). CFTR current was inhibited by 10 μM CFTR_inh_-172. Summary of CP7q dose-response data (right panel) (mean ± S.E., n = 3). (B) Summary of peak currents (mean ± S.E., n = 3). (C) Whole-cell CFTR Cl^-^ currents were recorded at a holding potential at 0 mV, and pulsing to voltages between ± 80 mV (in steps of 20 mV) in FRT cells expressing WT-CFTR (left panel). CFTR was slightly activated by 0.1 μM forskolin and potentiated by 10 μM CP7q. Current/voltage (I/V) plot of mean currents at the middle of each voltage pulse (middle panel). Summary of current density data measured at + 80 mV (mean ± S.E., n = 5, right panel). (D) Intracellular cAMP accumulation in FRT cells in response to addition of CP7q (10 μM) and forskolin (10 μM) (mean ± S.E., n = 4). (E) Effect of CP7q on cell viability 24 h after treatment as evaluated by MTS assays in HT-29 cells (mean ± S.E., n = 6). *P < 0.05, **P < 0.01.

Apical membrane currents were measured to verify potentiation activity of CP7q, the most potent compound, in WT-CFTR expressing FRT cells (Fig 3A). Apical membrane Cl^-^ current was measured in the FRT cells after basolateral membrane permeabilization with amphotericin B and in the presence of a transepithelial Cl^-^ gradient (apical, 64mM; basolateral, 129mM). Apical membrane current measurements in FRT-WT-CFTR cells gave an EC_50_ of ~ 0.88 μM for CP7q. The maximum efficacy of CP7q for CFTR potentiation was significantly higher than that of genistein when CFTR was stimulated by 0.1 μM forskolin in WT-CFTR expressing cells (Fig 3B). Whole-cell patch-clamp showed that CP7q strongly potentiated CFTR Cl^-^ current activated by 0.1 μM forskolin without altering the linear I/V relationship of activated CFTR in FRT cells expressing WT-CFTR (Fig 3C). CP7q did not activate WT- and ΔF508-CFTR without forskolin stimulation in whole cell patch-clamp recording, and CP7q-induced potentiation of CFTR Cl^-^ current was almost completely blocked by 10 μM CFTR_inh_-172 (S1 Fig). To test whether CP7q increases CFTR Cl^-^ channel activity via elevation of intracellular cAMP, we observed effect of CP7q in FRT cells and found out it did not affect the intracellular cAMP level (Fig 3D). CP7q thus potentiates CFTR without elevation of intracellular cAMP. To investigate the cytotoxic effect of CP7q, HT-29 cells were incubated with different concentrations of CP7q for 24 h and then cell viability was assessed using MTS assay. Concentration of CP7q up to 10 μM, where it showed maximum efficacy, showed no cytotoxicity (Fig 3E).

### CP7q Potentiates ΔF508- and G551D-CFTR Channel Activity

**Fig 4:**
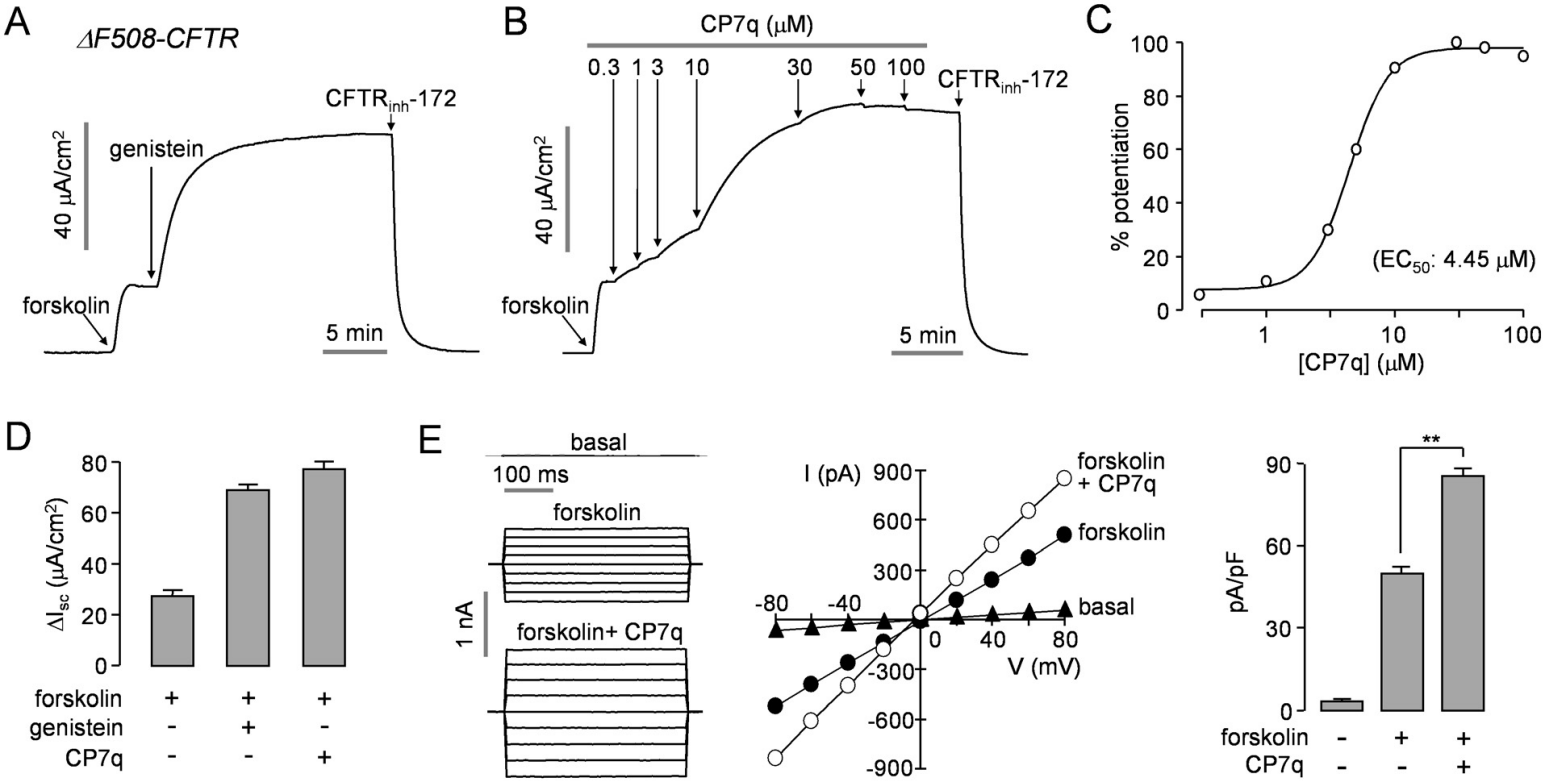
CP7q potentiates ΔF508-CFTR. Apical membrane currents were measured in FRT cells expressing human ΔF508-CFTR rescued by low temperature (27°C) incubation for 24 hours. (A, B) CFTR was activated by addition of 20 μM forskolin and potentiated with 50 μM genistein or different concentrations of CP7q. CFTR-dependent current was inhibited by 10 μM CFTR_inh_-172. (C) Summary of CP7q dose-response data (mean ± S.E., n = 3). (D) Summary of peak current (mean ± S.E., n = 3). (E) Whole-cell ΔF508-CFTR Cl^-^ currents were measured in FRT cells expressing ΔF508-CFTR rescued by low temperature (27°C) incubation for 24 hours (left panel). ΔF508-CFTR was activated by 10 μM forskolin and potentiated by 10 μM CP7q. Current/voltage (I/V) plot of mean currents at the middle of each voltage pulse (middle panel). Summary of current density data measured at + 80 mV (mean ± S.E., n = 5, right panel). **P < 0.01.

Apical membrane current measurements were performed to characterize CP7q potentiation activity in ΔF508-CFTR expressing FRT cells. ΔF508-CFTR was rescued by 24 h incubation at low temperature (27°C). CP7q significantly potentiated the ΔF508-CFTR Cl^-^ current activated by maximal cAMP stimulation in a dose-dependent manner with EC_50_ value of Δ4.45 μM, and the efficacy of CP7q was slightly higher than that of genistein (Fig 4A–4D). Whole-cell patch-clamp showed that CP7q significantly potentiated forskolin-induced Cl^-^ current without altering the linear I/V relationship of activated CFTR in FRT-ΔF508-CFTR cells (Fig 4E).

**Fig 5:**
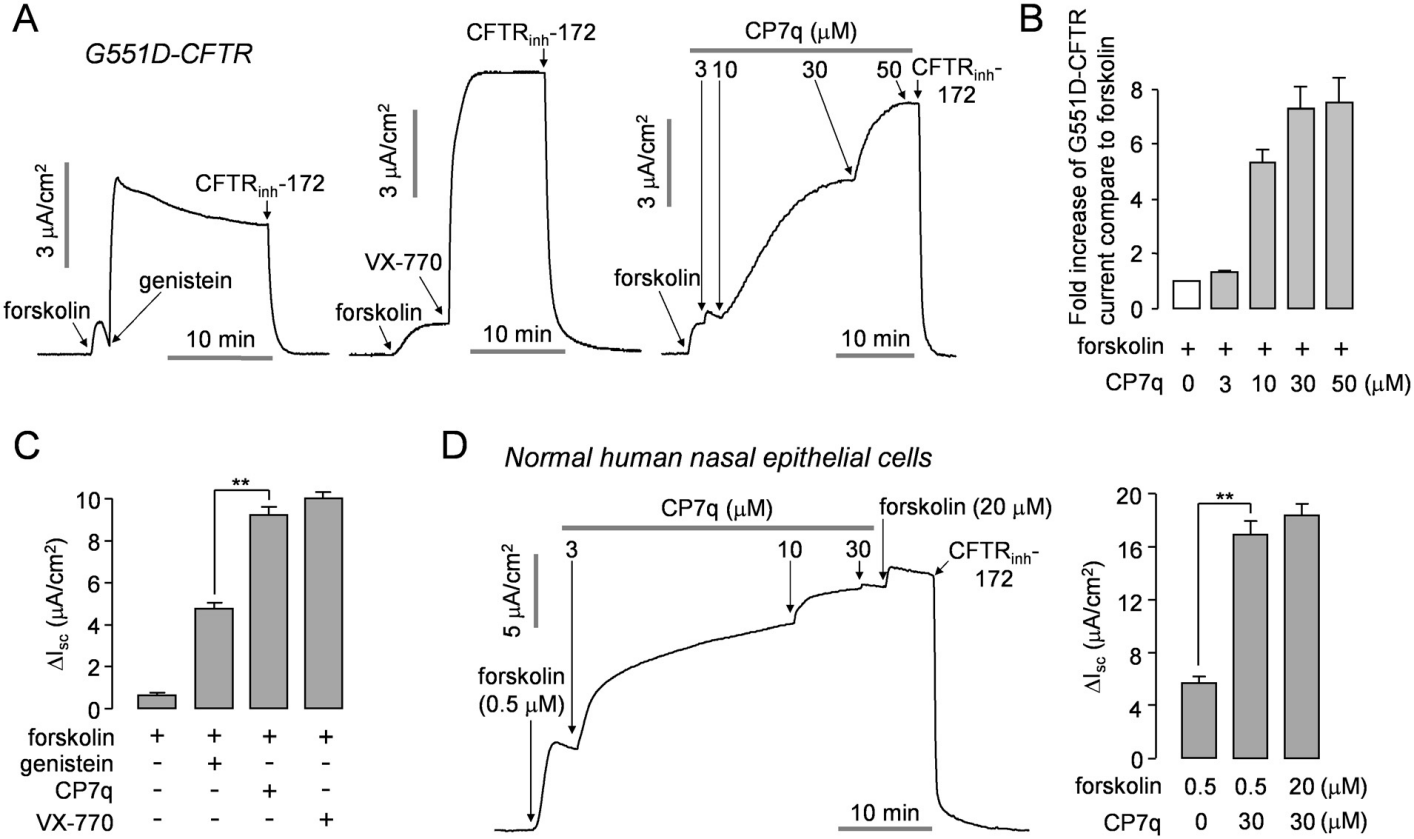
CP7q potentiates G551D-CFTR in FRT cells and WT-CFTR in primary cultured human nasal epithelial cells. (A) Apical membrane currents were measured in FRT cells expressing human G551D-CFTR. CFTR was stimulated by application of 20 μM forskolin and then 50 μM genistein (left panel), 10 μM VX-770 (middle panel) or indicated concentrations of CP7q (right panel) were applied to bath solution. CFTR-dependent current was inhibited by 10 μM CFTR_inh_-172. (B) Summary of CP7q-induced fold increase in G551D-CFTR current stimulated by 20 μM forskolin (mean ± S.E., n = 3). (C) Summary of 50 μM genistein, 10 μM VX-770 and 30 μM CP7q induced peak current (mean ± S.E., n = 3). (D) Short-circuit currents were measured in normal human nasal epithelial cells (left panel). CFTR was activated by 0.5 μM forskolin. CFTR was potentiated by indicated concentrations of CP7q and inhibited by 10 μM CFTR_inh_-172. ENaC was inhibited by 100 μM amiloride. Bar graph showing the summarized data of peak current (mean ± S.E., n = 3, right panel). **P < 0.01.

CP7q potentiation of the CF-causing gating mutant G551D-CFTR was further investigated. We measured apical membrane currents in FRT cells expressing G551D-CFTR to verify effect of CP7q on G551D-CFTR (Fig 5A–5C). Application of each of potentiators, genistein, VX-770, and CP7q significantly potentiated G551D-CFTR Cl^-^ current stimulated with maximal cAMP stimulation. Interestingly, CP7q potentiation of the G551D-CFTR Cl^-^ current activated by maximal forskolin was 1.9 times higher than that of genistein and comparable to VX-770.

### CP7q Potentiates CFTR Channel Activity in Human Airway Epithelium

To investigate whether CP7q potentiates CFTR in human primary cells, we measured short-circuit current in primary cultures of normal human nasal epithelial cells. In Fig 5D, CP7q remarkably potentiated CFTR Cl^-^ current activated by 0.5 μM forskolin in primary human airway epithelial cells. The potentiated CFTR Cl^-^ current was completely inhibited by 10 μM CFTR_inh_-172, a specific inhibitor of CFTR. ENaC was inhibited by pretreatment of amiloride to enhance the driving force for apical Cl^-^ secretion.

### CP7q Enhances Functional Rescue of ΔF508-CFTR by VX-809

**Fig 6:**
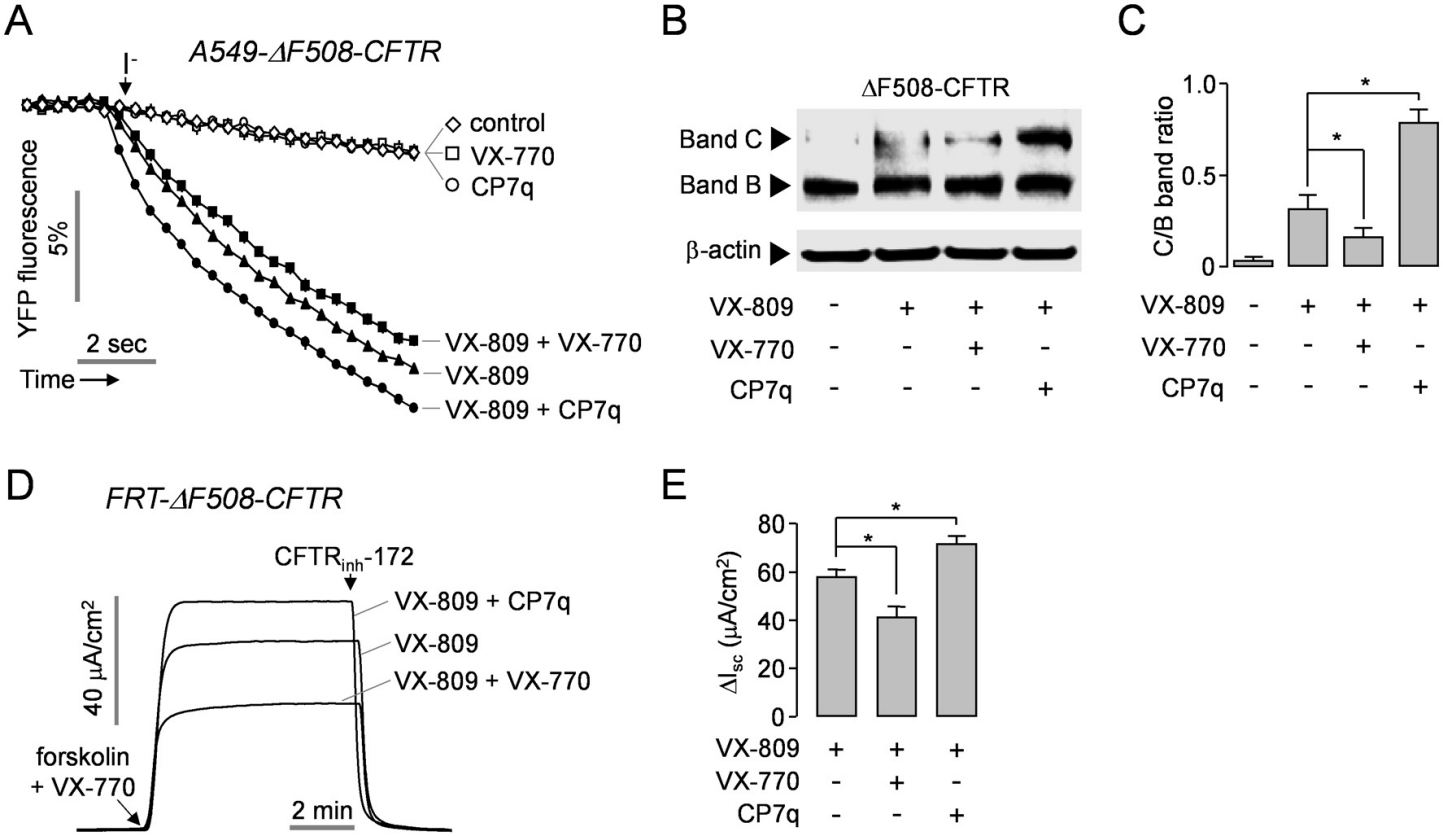
CP7q increases the functional rescue of ΔF508-CFTR by VX-809 in A549 and FRT cells. (A) ΔF508-CFTR expressing A549 cells were incubated with 5 μM VX-809 in the presence and absence of 10 μM CP7q or 5 μM VX-770 for 48 hours. The traces are showing iodide influx via the rescued ΔF508-CFTR stimulated with 10 μM forskolin and 10 μM VX-770 (mean ± S.E., n = 6). (B) Representative ΔF508-CFTR immunoblot at 48 h after treatment with 5 μM VX-809 in the presence and absence of 10 μM CP7q or 5 μM VX-770 in A549-ΔF508-CFTR cells. (C) The ratio of ΔF508-CFTR band C/B was summarized in bar graph (mean ± S.E., n = 3). (D) Representative apical membrane current traces showing effect of CP7q and VX-770 on VX-809-induced functional rescue of ΔF508-CFTR in FRT-ΔF508-CFTR cells. Well differentiated FRT cells were incubated with 5 μM VX-809 in the presence and absence of 10 μM CP7q or 5 μM VX-770 for 48 hours. ΔF508-CFTR currents were inhibited by 10 μM CFTR_inh_-172. (E) Bar graph showing the summarized data of peak current (mean ± S.E., n = 6). *P < 0.05.

To determine whether CP7q reduces the functional rescue of ΔF508-CFTR by VX-809 like VX-770 does, we observed the effect of CP7q on the VX-809-mediated rescue of ΔF508-CFTR. In Fig 6A, YFP quenching assay showed that the combined treatment with CP7q and VX-809 for 48 hours significantly increased the functional rescue of ΔF508-CFTR compared to VX-809 treatment alone, but the combined treatment with VX-770 and VX-809 decreased VX-809-mediated rescue of ΔF508-CFTR in A549 cells expressing ΔF508-CFTR. Immunoblot analysis revealed that the combined treatment with CP7q and VX-809 strongly increased the ratio of band C (mature glycosylated form) to band B (core-glycosylated, ER-retained protein) of ΔF508-CFTR compared to VX-809 treatment alone (Fig 6B and 6C). In addition, CP7q strongly increased apical membrane current of ΔF508-CFTR when combined with VX-809 while combined treatment with VX-809 and VX-770 decreased the apical membrane current in FRT cells expressing ΔF508-CFTR (Fig 6D and 6E).

## Discussion

Efforts aimed at the discovery and development of small-molecule modulators of CFTR as therapeutic agents for CF have produced some promising results such as Kalydeco (ivacaftor, VX-770) and Orkambi (lumacaftor/ivacaftor). Kalydeco showed remarkable clinical improvements in CF patients who have the G551D gating mutations [26, 27]. However, Orkambi targeting CF patients with the most common CF mutation, F508del, only offered a small improvement in lung function. Thus, there is a demand for development of novel correctors and potentiators, or more potent combinations of CFTR modulators for the clinical benefit of combination therapy in CF patients with F508del mutation.

Genistein enhances CFTR Cl^-^ current in various cell type and tissues, and the other flavonoids such as apigenin, kaempferol and quercetin also stimulate CFTR Cl^-^ current [21]. These flavonoids have a central heterocyclic ring and an aromatic substituent with hydroxy groups. Thus, to develop a new CFTR potentiator, we synthesized the hydroxypyrazolines with hydroxy groups like flavonoids and also with a benzenesulfonamide group which can easily participate in hydrogen bonding. In the present study, we revealed that CP7q, a hydroxypyrazoline, is a novel potentiator of CFTR which strongly potentiates WT-, ΔF508- and G551D-CFTR channel activity. Notably, we showed that CP7q potentiated 1.9 times more G551D-CFTR Cl^-^ current than genistein (Fig 5C). In this study, we could not evaluate the effect of CP7q on ΔF508- and G551D-CFTR channel activities in primary airway epithelial cells because of the limited access to primary cells derived from the CF patients. However, to investigate the effect of CP7q on CFTR in primary airway epithelial cells, we measured short-circuit current in primary cultures of normal human nasal epithelial cells and showed CP7q potently enhanced CFTR channel activity in a dose-dependent manner (Fig 5D). These results suggest that CP7q may exert its potentiator activity not only in cell lines but also in primary cells from CF patient.

Genistein and apigenin are postulated to interact directly to CFTR [43–45], and hypothesized to bind to the interface between nucleotide-binding domain 1(NBD1) and NBD2 [46]. VRT-532, a CFTR potentiator, directly binds to ΔF508- and G551D-CFTR and enhances CFTR Cl^-^ current by restoring its defective ATPase activity [47, 48]. In this study, we did not clearly reveal the mechanism of action of CP7q that enhances CFTR channel activity; however, even though we could not rule out the possibility that CP7q modulate any other protein that is involved in the regulatory mechanism of CFTR, the results from electrophysiological studies of CP7q, no effect on the cellular cAMP level, and structural and functional homology with the genistein, apigenin and VRT-532 suggest that CP7q may directly modulate CFTR channel activity.

Novel CFTR potentiators having a positive effect on corrector activity are still required to improve clinical benefit of combined therapy with corrector and potentiator in CF. In this study we investigated the effect of CP7q on the rescue of ΔF508-CFTR by VX-809 (Fig 6). Interestingly, CP7q significantly increased the mature glycosylated form and apical membrane current of ΔF508-CFTR when combined with VX-809 while combined treatment with VX-809 and VX-770 decreased the mature glycosylated form and the apical membrane current of ΔF508-CFTR compared to VX-809 treatment alone. These results indicate that the combined treatment with CP7q and VX-809 enhanced functional expression of ΔF508-CFTR along with potentiation of ΔF508-CFTR chloride channel currents. Therefore, the hydroxypyrazolines shown in this study may be useful as a source of novel chemical structure of potentiator for CF drug development.

In summary, a novel CFTR potentiator, CP7q, enhanced CFTR Cl^-^ current without alteration of cAMP level and cytotoxicity at the concentration showing maximum efficacy. CP7q enhanced ΔF508- and G551D-CFTR Cl^-^ current more potently than genistein. In addition, CP7q significantly increased wild type CFTR Cl^-^ current in primary cultures of human airway epithelial cells, and the combination treatment with CP7q and VX-809 significantly increased functional rescue of ΔF508-CFTR. Thus, CP7q may be useful for elucidating molecular mechanisms of CFTR modulation and as a potential CF drug development candidate.

## Supporting Information

S1 Fig Whole cell patch clamp recordings from FRT cells expressing WT-CFTR and ΔF508-CFTR. (TIF)

S1 File Experimental protocols of all synthesized compounds. (PDF)
