# Supplementary figures and images for: Potentiation of ΔF508- and G551D-CFTR-Mediated Cl- Current by Novel Hydroxypyrazolines
Source: PLoS One. 2016 Feb 10;11(2):e0149131. doi: 10.1371/journal.pone.0149131 (PMC4749168; doi:10.1371/journal.pone.0149131)

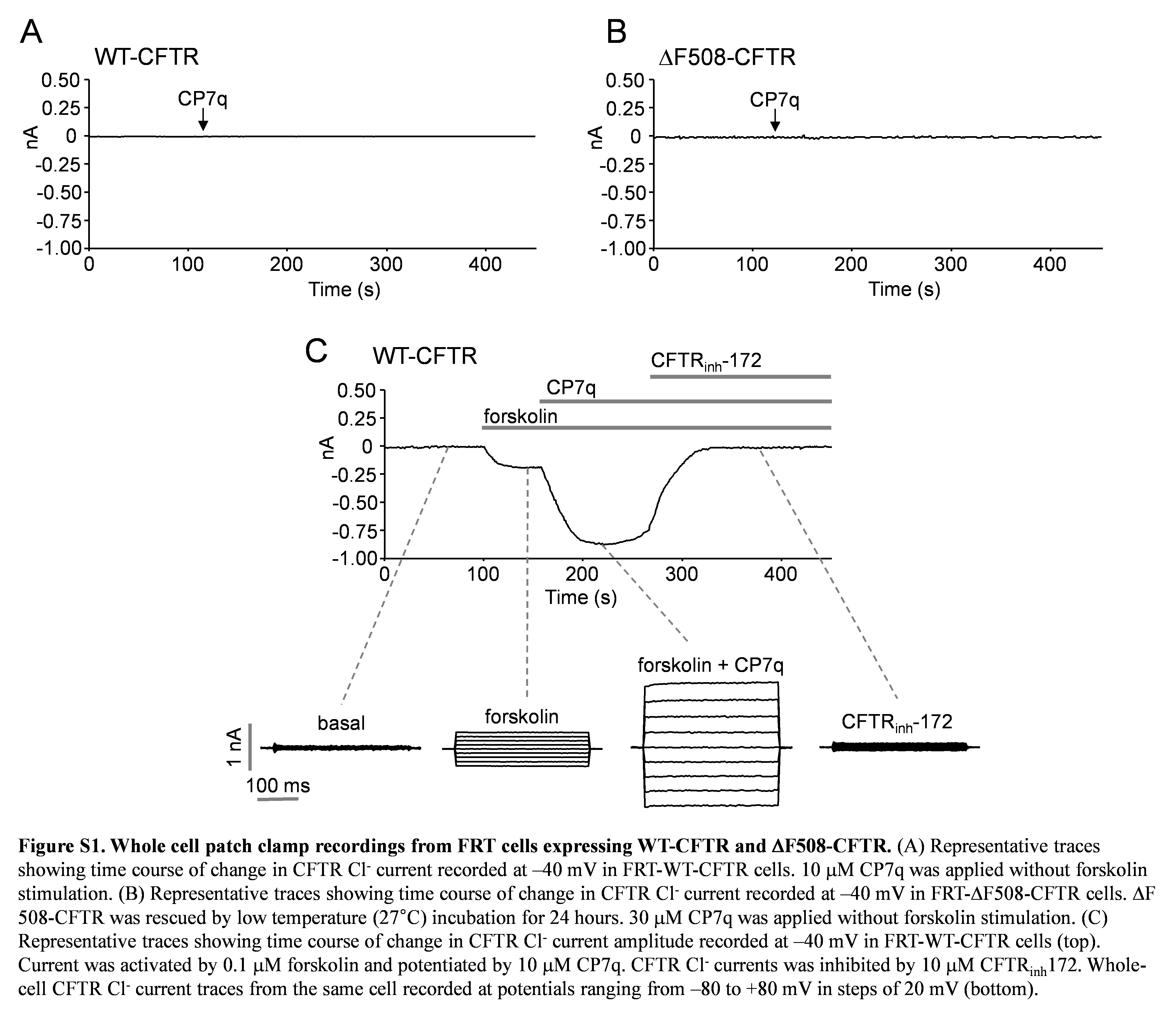

Supplement: S1 Fig — (TIF) [file pone.0149131.s001.tif]
