# Supplementary material for: Potentiation of ΔF508- and G551D-CFTR-Mediated Cl- Current by Novel Hydroxypyrazolines
Source: PLoS One. 2016 Feb 10;11(2):e0149131. doi: 10.1371/journal.pone.0149131 (PMC4749168; doi:10.1371/journal.pone.0149131)
Supplement: S1 File — (PDF) [file pone.0149131.s002.pdf]

Supporting Information

Experimental protocols

Melting points were determined in open capillaries in an electrical apparatus and are uncorrected. The IR spectra in KBr were recorded with ABB MB3000 DTGS IR instrument. ^{1}H NMR and ^{13}C NMR spectra were recorded on Bruker instrument at 300 MHz and 75.5 MHz respectively using DMSO-d_6 as a solvent. Chemical shifts are expressed in δ, ppm. Mass spectra (DART-MS) were recorded on a JEOL-AccuTOF JMS-T100LC Mass spectrometer having a DART (Direct Analysis in Real Time) source in ES^+ mode. The purity of the compounds was checked by ^{1}H NMR and thin layer chromatography (TLC) on silica gel plates using a mixture of petroleum ether and ethyl acetate as eluent. Thin layer chromatography (TLC) analyses were performed on aluminium silica gel sheets 60 F254 plates (Merck, Darmstadt, Germany) and spots were detected using a UV lamp at 254 nm. Abbreviations 's' for singlet, 'm' for multiplet, 'ex' for exchangeable proton are used for NMR assignments; 's' for strong and 'm' for medium for IR assignments.

Representative procedure for synthesis of protected hydroxy chalcones (5)

First of all, A mixture of 1e and 1f (1 mmol), MOMCl (2.5 mmol), and anhydrous K_2CO_3 (7 mmol) in dry acetone (20 mL) was well stirred under reflux for 1.5 h. After that filter the solution and evaporated the solution to afford the protected corresponding acetophenones. After that a stirred methanolic solution of NaOH (10 mmol) and appropriate acetophenones 4e and 4f (1 mol eq) at ice cold condition was added appropriate benzaldehydes 2 (1 mol eq) over 15 minute. The reaction mixture was stirred for 18 h at ambient temperature. The course of the reaction was monitored with TLC using ethyl acetate/petroleum ether as eluent. The contents of the flask were poured into crushed ice and neutralization with dil. HCl resulted in precipitation of the yellowish solid. The solid so obtained was filtered, washed with water and dried. The crude was crystallized from ethanol-chloroform (8:2) to afford the target protected chalcones 5 as crystalline compounds in excellent yield.

(E)-3-(2,3-dimethoxyphenyl)-1-[2-hydroxy-6-(methoxymethoxy)phenyl]-2-propen-1-one (5m)

M.p. 67-70 °C, yield 82%; IR (KBr, cm^{-1}): 1628 (s, C=O); ^{1}H NMR (DMSO-d_6, 300 MHz): δ 13.01 (s, 1H, ex, OH), 8.15 (d, 1H, *J* = 15.6 Hz, CH=<u>CH</u>), 7.95 (d, 1H, *J* = 15.6 Hz, <u>CH</u>=CH),7.36 (t, 1H, *J* = 7.8 Hz, Ar-H), 7.24 (m, 1H, Ar-H), 7.11 (t, 1H, *J* = 8.7 Hz, Ar-H), 6.98 (d, 1H, *J* = 7.8 Hz, Ar-H), 6.68 (dd, 2H, *J* = 8.1, 2.7 Hz, Ar-H), 5.31 (s, 2H, OCH_2), 3.92 (s, 6H, OCH_3), 3.54 (s, 3H, OCH_3); ^{13}C NMR (DMSO-d_6, 75.5 MHz): δ 193.7 (C=O), 164.2, 163.0, 158.1, 139.0, 135.2, 130.5, 124.2, 117.4, 113.0, 111.8, 104.5, 103.9, 97.4, 95.2, 56.6 (OCH_3), 55.5 (OCH_3), 55.5 (OCH_3).

(E)-3-(2,4-dimethoxyphenyl)-1-[2-hydroxy-6-(methoxymethoxy)phenyl]-2-propen-1-one (5n)

M.p. 102-104 °C, yield 83%; IR (KBr, cm^{-1}): 1628 (s, C=O); ^{1}H NMR (DMSO-d_6, 300 MHz): δ 13.01 (s, 1H, ex, OH), 8.17 (d, 1H, *J* = 15.6 Hz, CH=<u>CH</u>), 7.90 (d, 1H, *J* = 15.6 Hz, <u>CH</u>=CH),7.58 (d, 1H, *J* = 8.7 Hz, Ar-H), 7.33 (t, 1H, *J* = 8.1 Hz, Ar-H), 6.67 (d, 1H, *J* = 8.4 Hz, Ar-H), 6.62 (d, 1H, *J* = 8.4 Hz, Ar-H), 6.56 (d, 1H, *J* = 8.7 Hz, Ar-H), 6.49 (s, 1H, Ar-H), 5.30 (s, 2H, OCH_2), 3.92 (s, 6H, OCH_3), 3.54 (s, 3H, OCH_3); ^{13}C NMR (DMSO-d_6, 75.5 MHz): δ 194.7 (C=O), 164.3, 163.1, 158.2, 139.0, 135.2, 130.5, 125.2, 117.4, 113.0, 111.8, 105.5, 104.9, 98.4, 95.2, 56.6 (OCH_3), 55.5 (OCH_3), 55.5 (OCH_3).

(E)-3-(3,4-dimethoxyphenyl)-1-[2-hydroxy-6-(methoxymethoxy)phenyl]-2-propen-1-one (5o)

M.p. 70-72°C, yield 85%; IR (KBr, cm^{-1}): 1628 (s, C=O); ^{1}H NMR (DMSO-d_6, 300 MHz): δ 13.01 (s, 1H, ex, OH), 8.15 (d, 1H, *J* = 15.6 Hz, CH=<u>CH</u>), 7.95 (d, 1H, *J* = 15.6 Hz, <u>CH</u>=CH), 7.35 (t, 1H, *J* = 8.1 Hz, Ar-H), 7.24 (d, 1H, *J* = 8.4 Hz, Ar-H), 7.17 (m, 1H, Ar-H), 6.92 (d, 1H, *J* = 8.1 Hz, Ar-H), 6.68 (d, 1H, *J* = 8.4 Hz, Ar-H), 6.59 (d, 1H, *J* = 8.7 Hz, Ar-H), 5.32 (s, 2H, OCH_2), 3.95 (s, 6H, OCH_3), 3.55 (s, 3H, OCH_3); ^{13}C NMR (DMSO-d_6, 75.5 MHz): δ 194.2 (C=O), 164.4, 158.2, 151.4, 149.2, 143.3, 135.5, 128.2, 125.3, 123.1, 112.8, 111.8, 111.2, 110.2, 104.7, 95.3, 56.8 (OCH_3), 56.0 (OCH_3), 55.8 (OCH_3).

(E)-3-(2,3-dimethoxyphenyl)-1-[4-(hydroperoxymethoxy)-2-hydroxyphenyl]-2-propen-1-one (5p)

M.p. 79-80 °C, yield 80%; IR (KBr, cm^{-1}): 1628 (s, C=O); ^{1}H NMR (DMSO-d_6, 300 MHz): δ 13.01 (s, 1H, ex, OH), 8.15 (d, 1H, *J* = 15.6 Hz, CH=CH), 7.95 (d, 1H, *J* = 15.6 Hz, CH=CH), 7.35 (t, 1H, *J* = 8.1 Hz, Ar-H), 7.24 (d, 1H, *J* = 8.4 Hz, Ar-H), 7.15 (m, 1H, Ar-H), 6.92 (d, 1H, *J* = 8.1 Hz, Ar-H), 6.68 (d, 1H, *J* = 8.4 Hz, Ar-H), 6.59 (d, 1H, *J* = 8.7 Hz, Ar-H), 5.32 (s, 2H, OCH_2), 3.95 (s, 6H, OCH_3), 3.55 (s, 3H, OCH_3); ^{13}C NMR (DMSO-d_6, 75.5 MHz): δ 194.2 (C=O), 164.4, 158.2, 151.4, 149.2, 143.3, 135.5, 128.2, 125.3, 123.1, 112.8, 111.8, 111.2, 110.2, 104.7, 95.3, 56.8 (OCH_3), 56.0 (OCH_3), 55.8 (OCH_3).

(E)-3-(2,4-dimethoxyphenyl)-1-[4-(hydroperoxymethoxy)-2-hydroxyphenyl]-2-propen-1-one (5q)

M.p. 86-87 °C, yield 79%; IR (KBr, cm^{-1}): 1628 (s, C=O);^{1}H NMR (DMSO-d_6, 300 MHz): δ 13.53 (s, 1H, ex, OH), 8.15 (d, 1H, *J* = 15.6 Hz, CH=CH), 7.61 (d, 1H, *J* = 15.6 Hz, CH=CH), 7.37 (t, 1H, *J* = 8.1 Hz, Ar-H), 7.20 (d, 1H, *J* = 8.4 Hz, Ar-H), 7.17 (m, 1H, Ar-H), 6.92 (d, 1H, *J* = 8.1 Hz, Ar-H), 6.67 (d, 1H, *J* = 8.4 Hz, Ar-H), 6.58 (d, 1H, *J* = 8.7 Hz, Ar-H), 5.27 (s, 2H, OCH_2), 3.95 (s, 6H, OCH_3), 3.55 (s, 3H, OCH_3); ^{13}C NMR (DMSO-d_6, 75.5 MHz): δ 194.2 (C=O), 164.4, 158.2, 151.4, 149.2, 143.3, 135.5, 128.2, 125.3, 123.1, 112.8, 111.8, 111.2, 110.2, 104.7, 95.3, 56.8 (OCH_3), 56.0 (OCH_3), 55.8 (OCH_3).

(E)-3-(3,4-dimethoxyphenyl)-1-[2-hydroxy-4-(methoxymethoxy)phenyl]-2-propen-1-one (5r)

M.p. 80-82 °C, yield 78%; IR (KBr, cm^{-1}): 1628 (s, C=O);^{1}H NMR (DMSO-d_6, 300 MHz): δ 13.01 (s, 1H, ex, OH), 8.15 (d, 1H, *J* = 15.6 Hz, CH=CH), 7.95 (d, 1H, *J* = 15.6 Hz, CH=CH), 7.35 (t, 1H, *J* = 8.1 Hz, Ar-H), 7.24 (d, 1H, *J* = 8.4 Hz, Ar-H), 7.17 (m, 1H, Ar-H), 6.92 (d, 1H, *J* = 8.1 Hz, Ar-H), 6.68 (d, 1H, *J* = 8.4 Hz, Ar-H), 6.59 (d, 1H, *J* = 8.7 Hz, Ar-H), 5.32 (s, 2H, OCH_2), 3.95 (s, 6H, OCH_3), 3.55 (s, 3H, OCH_3); ^{13}C NMR (DMSO-d_6, 75.5 MHz): δ 194.2 (C=O), 163.4, 159.2, 151.4, 148.2, 143.3, 134.5, 127.2, 125.3, 123.1, 112.7, 110.8, 111.2, 110.2, 103.7, 95.3, 56.8 (OCH_3), 56.0 (OCH_3), 55.8 (OCH_3).

Representative procedure for synthesis of hydroxychalcones 3a-3l

Hydroxychalcones (3a-3l) from acetophenones (1a-1d)

To a stirred methanolic solution of NaOH (10 mmol) and appropriate acetophenones 1(10 mmol) at ice cold condition was added appropriate benzaldehydes 2 (1 mol eq) over 15 minute. The reaction mixture was stirred for 18 hour at ambient temperature. The course of the reaction was monitored on TLC using ethyl acetate/petroleum ether as eluent. The contents of the flask were poured onto crushed ice and neutralization with dil. HCl resulted in precipitation of the yellowish solid. The solid so obtained was filtered, washed with water and dried. The crude was crystallized from ethanol-chloroform (8:2) to afford the target chalcones 3a-3l as crystalline compounds in excellent yield.

Hydroxychalcones (3m-3r) from protected chalcones (5m-5r)

A solution of protected chalcones (5m-5r;1 mol eq) in 3 N HCl were refluxed for 1-3 h. The course of the reaction was monitored on TLC using ethyl acetate/petroleum ether as eluent. Reaction mixtures were poured into crushed ice and resulted in precipitation of the solid. The solid so obtained was filtered, washed with water and dried. The crude was crystallized from ethanol-chloroform (8:2) to afford the target chalcones 3m-3r in good yield.

(E)-3-(2,3-dimethoxyphenyl)-1-(2-hydroxyphenyl)-2-propen-1-one (3a) [1]

M.p. 80-82 °C, yield 82%; IR (KBr, cm^{-1}): 1628 (s, C=O stretch); ^{1}H NMR (DMSO-d_6, 300 MHz): δ 13.10 (brs, ex, 1H, OH), 8.22 (d, 1H, *J* = 15.6 Hz, C<u>H</u>= CH-CO), 7.93 (d, 1H, *J* = 8.1 Hz, Ar-H), 7.77 (d, 1H, *J* = 15.6 Hz, CH= C<u>H</u>-CO), 7.51 (t, 1H, *J* = 7.8 Hz, Ar-H), 7.70 (d, 1H, *J* = 8.1 Hz, Ar-H), 7.13 (t, 1H, *J* = 7.8 Hz, Ar-H), 7.07-6.98 (m, 2H, Ar-H), 6.95 (d, 1H, *J* = 7.8 Hz, Ar-H), 3.94 (s, 3H, OCH_3), 3.91 (s, 3H, OCH_3); ^{13}C NMR (DMSO-d_6, 75.5 MHz): δ 194.1 (C=O), 163.6, 153.3, 149.2, 140.4, 136.2, 129.6, 128.8, 124.2, 121.7, 120.1, 119.9, 118.7, 118.6, 114.6, 61.3 (OCH_3), 55.9 (OCH_3).

(E)-3-(2,4-dimethoxyphenyl)-1-(2-hydroxyphenyl)-2-propen-1-one (3b)

M.p. 92-94 °C (Lit. [2] 88-89 °C), yield 82%; IR (KBr, cm^{-1}): 1628 (s, C=O stretch); ^{1}H NMR (DMSO-d_6, 300 MHz): δ 13.10 (brs, ex, 1H, OH), 8.22 (d, 1H, *J* = 15.6 Hz, C<u>H</u>= CH-CO), 7.93

(d, 1H, *J* = 8.1 Hz, Ar-H), 7.77 (d, 1H, *J* = 15.6 Hz, CH= C<u>H</u>-CO), 7.51 (t, 1H, *J* = 7.8 Hz, Ar-H), 7.70 (d, 1H, *J* = 8.1 Hz, Ar-H), 7.13 (t, 1H, *J* = 7.8 Hz, Ar-H), 7.06-6.97 (m, 2H, Ar-H), 6.93 (d, 1H, *J* = 7.8 Hz, Ar-H), 3.94 (s, 3H, OCH_3), 3.91 (s, 3H, OCH_3). ^{13}C NMR (DMSO-d_6, 75.5 MHz): δ 194.2 (C=O), 163.5, 153.2, 149.2, 140.4, 135.2, 129.6, 128.8, 125.2, 121.7, 120.2, 119.9, 118.6, 118.6, 113.6, 61.2 (OCH_3), 55.8 (OCH_3).

(E)-3-(3,4-dimethoxyphenyl)-1-(2-hydroxyphenyl)-2-propen-1-one (3c) [3]

M.p. 90-92 °C, yield 80%; IR (KBr, cm^{-1}): 1628 (s, C=O stretch); ^{1}H NMR (DMSO-d_6, 300 MHz): δ 12.93 (brs, ex, 1H, OH), 7.94 (d, 2H, *J* = 8.4 Hz, Ar-H), 7.87 (d, 1H, *J* = 15.3 Hz, C<u>H</u>= CH-CO), 7.70 (s, 1H, Ar-H), 7.53 (d, 1H, *J* = 15.6 Hz, CH= C<u>H</u>-CO), 7.03 (d, 2H, *J* = 8.1 Hz, Ar-H), 6.93 (d, 2H, *J* = 8.4 Hz, Ar-H), 3.98 (s, 3H, OCH_3), 3.95 (s, 3H, OCH_3). ^{13}C NMR (DMSO-d_6, 75.5 MHz): δ 194.3 (C=O), 163.4, 153.2, 149.2, 140.4, 136.2, 128.6, 128.7, 124.1, 121.6, 120.2, 119.9, 118.7, 118.6, 114.6, 61.2 (OCH_3), 55.6 (OCH_3).

(E)-3-(2,3-dimethoxyphenyl)-1-(2-hydroxy-6-methoxyphenyl)-2-propen-1-one (3d)

M.p. 90-92 °C, yield 78%; IR (KBr, cm^{-1}): 1628 (s, C=O stretch); ^{1}H NMR (DMSO-d_6, 300 MHz): δ 13.20 (brs, ex, 1H, OH), 8.12 (d, 1H, *J* = 15.6 Hz, C<u>H</u>= CH-CO), 7.94 (d, 1H, *J* = 15.6 Hz, CH= C<u>H</u>-CO), 7.37 (t, 1H, *J* = 8.4 Hz, Ar-H), 7.26 (d, 1H, *J* = 8.1 Hz, Ar-H), 7.10 (t, 1H, *J* = 8.1 Hz, Ar-H), 6.98 (d, 1H, *J* = 8.1 Hz, Ar-H), 6.63 (d, 1H, *J* = 8.7 Hz, Ar-H), 6.64 (d, 1H, *J* = 8.1 Hz, Ar-H), 3.95 (s, 3H, OCH_3), 3.91 (s, 6H, 2x OCH_3); ^{13}C NMR (DMSO-d_6, 75.5 MHz): δ 194.7, 164.8, 161.0, 153.2, 149.0, 137.8, 136.0, 135.8, 129.5, 128.9, 124.1, 119.8, 114.0, 112.1, 110.9, 101.5, 61.32 (OCH_3), 55.92 ($2OCH_3$).

(E)-3-(2,4-dimethoxyphenyl)-1-(2-hydroxy-6-methoxyphenyl)-2-propen-1-one (3e)

M.p. 98-100 °C, yield 74%; IR (KBr, cm^{-1}): 1628 (s, C=O stretch); ^{1}H NMR (DMSO-d_6, 300 MHz): δ 13.25 (brs, ex, 1H, OH), 8.15 (d, 1H, *J* = 15.6 Hz, C<u>H</u>= CH-CO), 7.89 (d, 1H, *J* = 15.6 Hz, CH= C<u>H</u>-CO), 7.57 (d, 1H, *J* = 8.4 Hz, Ar-H), 7.35 (t, 1H, *J* = 8.1 Hz, Ar-H), 6.62 (d, 1H, *J* = 8.7 Hz, Ar-H), 6.56 (d, 1H, *J* = 9.0 Hz, Ar-H), 6.49-6.45 (m, 1H, Ar-H), 6.40 (d, 1H, *J*= 9.0 Hz, Ar-H), 3.95 (s, 3H, OCH_3), 3.91 (s, 3H, OCH_3), 3.89 (s, 3H, OCH_3); ^{13}C NMR (DMSO-d_6, 75.5

MHz): δ 194.7, 164.8, 161.5, 160.9, 160.4, 138.9, 136.0, 130.6, 125.4, 112.2, 111.3, 110.9, 110.7, 101.5, 101.1, 55.8 (OCH_3), 55.6 (OCH_3), 55.4 (OCH_3).

(E)-3-(3,4-dimethoxyphenyl)-1-(2-hydroxy-6-methoxyphenyl)-2-propen-1-one (3f) [7]

M.p. 100-102 °C, yield 76%; IR (KBr, cm^{-1}): 1628 (s, C=O stretch); ^1H NMR (DMSO-d_6, 300 MHz): δ 13.24 (brs, ex, 1H, OH), 7.82 (d, 1H, *J* = 15.6 Hz, C<u>H</u>= CH-CO), 7.76 (d, 1H, *J* = 15.6 Hz, CH= C<u>H</u>-CO), 7.38 (t, 1H, *J* = 8.4 Hz, Ar-H), 7.25 (d, 1H, *J* = 8.7 Hz, Ar-H), 7.15 (s, 1H, Ar-H), 6.92 (d, 1H, *J* = 8.4 Hz, Ar-H), 6.63 (d, 1H, *J* = 8.4 Hz, Ar-H), 6.45 (d, 1H, *J* = 8.1 Hz, Ar-H), 3.96 (s, 9H, 3x OCH_3); ^{13}C NMR (DMSO-d_6, 75.5 MHz): δ 194.2, 164.8, 160.9, 151.3, 149.2, 143.2, 135.6, 128.4, 125.5, 122.8, 112.1, 111.2, 111.0, 101.5, 56.0 (OCH_3), 55.9 (OCH_3).

(E)-3-(2,3-dimethoxyphenyl)-1-(2-hydroxy-5-methylphenyl)-2-propen-1-one (3g)

M.p 74-76 °C, yield 75%; IR (KBr, cm^{-1}):1628 (s, C=O); ^1H NMR (DMSO-d_6, 300 MHz): δ 12.98 (s, 1H, ex, OH), 8.21 (d, 1H, *J* = 15.6 Hz, CH=<u>CH</u>), 7.70 (d, 1H, *J* = 15.3 Hz, <u>CH</u>=CH), 7.70 (s, 1H, Ar-H), 7.33 (d, 1H, *J* = 7.2 Hz, Ar-H), 7.28 (s, 1H, Ar-H), 7.14 (t, *J* = 8.1 Hz, Ar-H), 7.01 (d, 1H, *J* = 8.1 Hz, Ar-H), 6.96 (d, 1H, *J* = 8.4 Hz, Ar-H), 3.94 (s, 3H, OCH_3), 3.85 (s, 3H, OCH_3), 2.37 (s, 3H, CH_3). ^{13}C NMR (DMSO-d_6, 75.5 MHz): δ 194.1, 164.6, 160.9, 150.3, 149.2, 143.2, 134.6, 127.4, 124.5, 121.8, 112.2, 111.1, 110.0, 101.5, 56.0 (OCH_3), 55.8 (OCH_3). 21.3 (CH_3)

(E)-3-(2,4-dimethoxyphenyl)-1-(2-hydroxy-5-methylphenyl)-2-propen-1-one (3h)

M.p 110-112 °C, yield 73%; IR (KBr, cm^{-1}): 1628 (s, C=O); ^1H NMR (DMSO-d_6, 300 MHz): δ 13.01 (s, 1H, ex, OH), 8.20 (d, 1H, *J* = 15.6 Hz, CH=<u>CH</u>), 7.80 (d, 1H, *J* = 8.4 Hz, Ar-H), 7.68 (d, 1H, *J* = 15.6 Hz, <u>CH</u>=CH),7.30 (d, 1H, *J* = 8.7 Hz, Ar-H), 6.93 (d, 1H, *J* = 8.7 Hz, Ar-H), 6.57 (d, 1H, *J* = 8.4 Hz, Ar-H), 6.51 (s, 1H, Ar-H), 3.95 (s, 3H, OCH_3), 3.91 (s, 3H, OCH_3), 2.37 (s, 3H, CH_3); ^{13}C NMR (DMSO-d_6, 75.5 MHz): δ 194.2, 164.8, 160.9, 151.3, 149.2, 143.2, 135.6, 128.4, 125.5, 122.8, 112.1, 111.2, 111.0, 101.5, 56.0 (OCH_3), 55.9 (OCH_3). 21.2 (CH_3).

(E)-3-(3,4-dimethoxyphenyl)-1-(2-hydroxy-5-methylphenyl)-2-propen-1-one (3i)

M.p. 110-113 ℃ (Lit. [4] 121-123 ℃), yield 76%; IR (KBr, cm^{-1}): 1628 (s, C=O);^{1}H NMR (DMSO-d_6, 300 MHz): δ 13.01 (s, 1H, ex, OH), 7.88 (d, 1H, *J* = 15.3 Hz, CH=<u>CH</u>), 7.70 (s, 1H, Ar-H), 7.52 (d, 1H, *J* = 15.0 Hz, <u>CH</u>=CH),7.30 (t, 2H, Ar-H), 7.19 (s, 1H, Ar-H), 6.95-6.91 (m, 2H, Ar-H), 3.91 (s, 3H, OCH_3), 3.82 (s, 3H, OCH_3), 2.37 (s, 3H, CH_3); ^{13}C NMR (DMSO-d_6, 75.5 MHz): δ 194.2 (C=O), 164.8, 160.5, 151.2, 148.2, 142.2, 135.6, 127.4, 125.5, 122.8, 112.1, 111.2, 111.0, 101.4, 56.1 (OCH_3), 55.9 (OCH_3). 21.2 (CH_3).

(E)-1-(5-chloro-2-hydroxyphenyl)-3-(2,3-dimethoxyphenyl)-2-propen-1-one (3j)

M.p. 102-104 ℃, yield 75%; IR (KBr, cm^{-1}): 1628 (s, C=O);^{1}H NMR (DMSO-d_6, 300 MHz): δ 13.01 (s, 1H, ex, OH), 8.21 (d, 1H, *J* = 15.6 Hz, CH=<u>CH</u>), 7.17 (d, 1H, *J* = 15.6 Hz, <u>CH</u>=CH), 7.62 (d, 1H, *J* = 7.5 Hz, Ar-H), 7.58 (m, 1H, Ar-H), 7.34 (d, 1H, *J* = 5.4 Hz, Ar-H), 6.98 (d, 1H, *J* = 9.0 Hz, Ar-H), 6.59 (d, 1H, *J* = 6.9 Hz, Ar-H), 6.50 (s, 1H, Ar-H), 3.95 (s, 3H, OCH_3), 3.90 (s, 3H, OCH_3); ^{13}C NMR (DMSO-d_6, 75.5 MHz): δ 194.1, 164.8, 160.9, 151.3, 149.2, 143.1, 135.5, 128.4, 124.5, 121.8, 112.1, 111.1, 111.0, 101.4, 56.0 (OCH_3), 55.8 (OCH_3).

(E)-1-(5-chloro-2-hydroxyphenyl)-3-(2,4-dimethoxyphenyl)-2-propen-1-one (3k)

M.p. 98-100 ℃, yield 74%; IR (KBr, cm^{-1}): (s, C=O);^{1}H NMR (DMSO-d_6, 300 MHz): δ 13.01 (s, 1H, ex, OH), 8.22 (d, 1H, *J* = 15.6 Hz, CH=<u>CH</u>), 7.18 (d, 1H, *J* = 15.6 Hz, <u>CH</u>=CH), 7.63 (d, 1H, *J* = 7.5 Hz, Ar-H), 7.59 (m, 1H, Ar-H), 7.34 (d, 1H, *J* = 5.4 Hz, Ar-H), 6.98 (d, 1H, *J* = 9.0 Hz, Ar-H), 6.59 (d, 1H, *J* = 6.9 Hz, Ar-H), 6.51 (s, 1H, Ar-H), 3.96 (s, 3H, OCH_3), 3.90 (s, 3H, OCH_3). ^{13}C NMR (DMSO-d_6, 75.5 MHz): δ 194.3 (C=O), 164.6, 160.8, 151.3, 148.2, 143.1, 135.7, 128.4, 125.5, 122.8, 112.1, 111.2, 111.0, 101.5, 56.0 (OCH_3), 55.9 (OCH_3).

(*E*)-1-(5-chloro-2-hydroxyphenyl)-3-(3,4-dimethoxyphenyl)-2-propen-1-one (3l)

M.p. 158-160 ℃ (Lit. [5] 102-103 ℃), yield 75%; IR (KBr, cm^{-1}): 1628 (s, C=O); ^{1}H NMR (DMSO-d_6, 300 MHz):δ 13.01 (s, 1H, ex, OH), 8.03 (s, 1H, Ar-H), 7.93 (d, 1H, *J* = 16.8 Hz, CH=<u>CH</u>), 7.41 (d, 1H, *J* = 15.6 Hz, <u>CH</u>=CH),7.45 (d, 1H, *J* = 8.4 Hz, Ar-H), 7.31 (d, 1H, *J* = 8.4 Hz, Ar-H), 7.28 (d, 1H, *J* = 8.7 Hz, Ar-H), 7.01 (d, 1H, *J* = 8.7 Hz, Ar-H), 6.95 (d, 1H, *J* = 8.4 Hz, Ar-H), 4.01 (s, 3H, OCH_3), 3.97 (s, 3H, OCH_3); ^{13}C NMR (DMSO-d_6, 75.5 MHz): δ 194.5

(C=O), 164.7, 160.8, 151.3, 149.2, 142.2, 135.5, 128.4, 125.4, 122.7, 112.1, 111.2, 111.0, 101.5, 56.1 (OCH_3), 55.8 (OCH_3).

(E)-1-(2,6-dihydroxyphenyl)-3-(2,3-dimethoxyphenyl)-2-propen-1-one (3m)

M.p. 138-140 °C, yield 82%; IR (KBr, cm^{-1}): 1636 (s, C=O stretch); ^{1}H NMR (DMSO-d_6, 300 MHz): δ 13.20 (brs, ex, 1H, OH), 9.86 (brs, ex, 1H, OH), 8.17 (d, 1H, *J* = 15.6 Hz, C<u>H</u>= CH-CO), 8.09 (d, 1H, *J* = 15.6 Hz, CH= C<u>H</u>-CO), 7.28-7.26 (m, 2H, Ar-H), 7.09 (t, 1H, *J* = 7.8 Hz, Ar-H), 6.97 (d, 1H, *J* = 7.8 Hz. Ar-H), 6.45 (d, 2H, *J* = 8.1 Hz, Ar-H), 3.90 (s, 6H, OCH_3); ^{13}C NMR (DMSO-d_6, 75.5 MHz): δ 194.6 (C=O), 161.3, 153.1, 148.9, 138.4, 135.9, 129.3, 128.5, 124.1, 119.91, 114.1, 110.9, 108.4, 61.4 (OCH_3), 55.9 (OCH_3).

(E)-1-(2,6-dihydroxyphenyl)-3-(2,4-dimethoxyphenyl)-2-propen-1-one (3n)

M.p. 102-104 °C, yield 84%; IR (KBr, cm^{-1}): 1636 (s, C=O stretch); ^{1}H NMR (DMSO-d_6, 300 MHz): δ 13.20 (brs, ex, 1H, OH), 9.86 (brs, ex, 1H, OH), 8.17 (d, 1H, *J* = 15.6 Hz, C<u>H</u>= CH-CO), 8.09 (d, 1H, *J* = 15.6 Hz, CH= C<u>H</u>-CO), 7.29-7.26 (m, 2H, Ar-H), 7.07 (t, 1H, *J* = 7.8 Hz, Ar-H), 6.95 (d, 1H, *J* = 7.8 Hz. Ar-H), 6.44 (d, 2H, *J* = 8.1 Hz, Ar-H), 3.90 (s, 6H, OCH_3). δ 194.6 (C=O), 161.3, 153.1, 148.9, 138.4, 135.9, 129.3, 128.5, 124.1, 119.9, 114.1, 110.9, 108.4, 61.4 (OCH_3), 55.9 (OCH_3).

(E)-1-(2,6-dihydroxyphenyl)-3-(3,4-dimethoxyphenyl)-2-propen-1-one (3o)

M.p. 136-138 °C (Lit. [6] 140 °C), yield 70%; IR (KBr, cm^{-1}): 1636 (s, C=O stretch); ^{1}H NMR (DMSO-d_6, 300 MHz): δ 13.20 (brs, ex, 1H, OH), 9.86 (brs, ex, 1H, OH), 8.11 (d, 1H, *J* = 15.6 Hz, C<u>H</u>= CH-CO), 7.95 (d, 1H, *J* = 15.6 Hz, CH= C<u>H</u>-CO), 7.30-7.24 (m, 2H, Ar-H), 7.09 (t, 1H, *J* = 7.8 Hz, Ar-H), 6.97 (d, 1H, *J* = 7.8 Hz. Ar-H), 6.42 (d, 2H, *J* = 8.1 Hz, Ar-H), 3.90 (s, 6H, OCH_3). δ 194.5 (C=O), 161.2, 153.1, 148.9, 137.4, 135.8, 129.2, 128.4, 124.0, 119.9, 113.1, 110.7, 107.2, 61.2 (OCH_3), 55.8 (OCH_3).

(E)-1-(2,4-dihydroxyphenyl)-3-(2,3-dimethoxyphenyl)-2-propen-1-one (3p)

M.p 178-180 °C (Lit. [7] 188-189 °C), yield 74%; IR (KBr, cm^{-1}): 1630 (s, C=O); ^{1}H NMR (DMSO-d_6, 300 MHz): δ 13.32 (brs, ex, 1H, OH), 8.18 (d, 1H, *J* = 15.6 Hz, C<u>H</u>= CH-CO), 7.84

(d, 1H, *J* = 9.0 Hz, Ar-H), 7.68 (d, 1H, *J* = 15.6 Hz, CH = C<u>H</u>-CO), 7.11 (m, 2H, Ar-H), 7.00 (d, 1H, *J* = 7.5 Hz, Ar-H), 6.44 (s, 2H, Ar-H), 3.93 (s, 6H, OCH_3); ^{13}C NMR (DMSO-d_6, 75.5 MHz): δ 194.5 (C=O), 161.2, 153.1, 148.9, 137.4, 135.8, 129.2, 128.4, 124.0, 119.9, 113.1, 110.7, 107.2, 61.2 (OCH_3), 55.8 (OCH_3).

(E)-1-(2,4-dihydroxyphenyl)-3-(2,4-dimethoxyphenyl)-2-propen-1-one (3q) [8]
M.p 178-180 °C, yield 72%; IR (KBr, cm^{-1}): 1628 (s, C=O); ^{1}H NMR (DMSO-d_6, 300 MHz): δ 13.31 (brs, ex, 1H, OH), 7.85 (d, 1H, *J* = 15.6 Hz, C<u>H</u>= CH-CO), 7.82 (s, 1H, Ar-H), 7.43 (d, 1H, *J* = 15.6 Hz, CH= C<u>H</u>-CO), 7.17 (s, 1H, Ar-H), 6.92 (d, 1H, *J* = 7.8 Hz, Ar-H), 6.46-6.38 (m, 3H, Ar-H), 3.97 (s, 3H, OCH_3), 3.95 (s, 3H, OCH_3); ^{13}C NMR (DMSO-d_6, 75.5 MHz): δ 194.2 (C=O), 161.2, 153.1, 148.9, 137.4, 135.8, 129.2, 128.4, 124.0, 119.9, 113.1, 110.7, 107.2, 61.2 (OCH_3), 55.8 (OCH_3).

(E)-1-(2,4-dihydroxyphenyl)-3-(3,4-dimethoxyphenyl)-2-propen-1-one (3r) [9]
M.p 178-180 °C, yield 76%; IR (KBr, cm^{-1}): 1630 (s, C=O); ^{1}H NMR (DMSO-d_6, 300 MHz): δ 13.30 (brs, ex, 1H, OH), 7.84 (d, 1H, *J* = 15.6 Hz, C<u>H</u>= CH-CO), 7.81 (s, 1H, Ar-H), 7.43 (d, 1H, *J* = 15.6 Hz, CH= C<u>H</u>-CO), 7.17 (s, 1H, Ar-H), 6.92 (d, 1H, *J* = 7.8 Hz, Ar-H), 6.49-6.37 (m, 3H, Ar-H), 3.97 (s, 3H, OCH_3), 3.94 (s, 3H, OCH_3); ^{13}C NMR (DMSO-d_6, 75.5 MHz): δ 194.3 (C=O), 160.2, 153.2, 148.9, 137.4, 134.4, 129.1, 128.2, 123.0, 110.9, 112.1, 110.7, 106.2, 61.1 (OCH_3), 54.8 (OCH_3).

General procedure of synthesis of hydroxypyrazolines (7a-7r)
Ethanolic solution of chalcones 3, (1.00 mmol) and 4-hydrazinobenzenesulfonamide hydrochloride (6), (1.10 mmol) in presence of catalytic amount of glacial acetic acid was refluxed for 8-12 h. The course of the reaction was monitored by TLC. The contents of reaction flask were concentrated and left overnight resulting in the formation of crystals. The solid so obtained was filtered, dried over vacuum pump and crystallized from ethanol to afford the target pyrazolines 7 as fluffy colorless crystals in excellent yield.

4-[5-(2,3-dimethoxy phenyl)-3-(2-hydroxyphenyl)-4,5-dihydro-1H-pyrazol-1-yl]benzenesulfonamide (7a)

M.p. 190-192 °C, yield 82%; IR (KBr, cm^{-1}): 3325, 3248 (m, N-H stretch), 1589 (s, C=N stretch), 1512 (s, N-H bend), 1327 and 1165 (s, SO_2 stretch); ^1H NMR (DMSO-d_6, 300 MHz): δ^1H NMR (DMSO-d_6, 300 MHz): δ 10.35 (br s, ex, 1H, OH), 7.63 (d, 2H, *J* = 8.7 Hz, Ar-H), 7.51 (d, 1H, *J* = 7.5 Hz, Ar-H), 7.33-7.28 (m, 1H, Ar-H), 7.04 (s, ex, 2H, SO_2NH_2), 6.91 (d, 1H, *J* = 7.2 Hz, Ar-H), 6.66-6.63 (m, 1H, Ar-H), 5.70 (dd, *J* =12.6, 5.6 Hz, 1H, pyrazoline-5H), 4.11 (dd, *J* = 18.0, 12.6 Hz, 1H, pyrazoline-4H), 3.86 (s, 3H, OCH_3), 3.81 (s, 3H, OCH_3), 3.32 ((dd, *J* = 18.0, 5.6 Hz, 1H, pyrazoline-4H)); ^{13}C NMR (DMSO-d_6, 75.5 MHz): δ 156.5, 153.1, 152.7, 146.2, 145.6, 134.5, 133.3, 131.4, 128.7, 127.7, 125.0, 120.3, 118.7, 116.6, 113.1, 112.1, 60.7 (C5-pyrazoline), 57.4 (OCH_3), 56.1 (OCH_3), 43.5 (C4-pyrazoline); DART MS: m/z 452.34 $[M+H]^+$, $C_{23}H_{23}N_3O_5SH^+$ Calcd. 452.510.

4-[5-(2,4-dimethoxyphenyl)-3-(2-hydroxyphenyl)-4,5-dihydro-1H-pyrazol-1-yl]benzenesulfonamide (7b)

M.p. 224-226 °C, yield 84%; IR (KBr, cm^{-1}): 3340, 3248 (m, N-H stretch), 1597 (s, C=N stretch), 1497 (s, N-H bend), 1327 and 1142 (s, SO_2 stretch); ^1H NMR (DMSO-d_6, 300 MHz): δ 10.36 (br s, ex, 1H, OH),7.62 (d, 2H, *J* = 8.7 Hz, Ar-H), 7.49 (d, 1H, *J* = 7.8 Hz, Ar-H), 7.32-7.27 (m, 1H, Ar-H), 7.04 (s, ex, 2H, SO_2NH_2), 7.00-6.89 (m, 4H, Ar-H), 6.64 (d, 1H, *J* = 2.4 Hz, Ar-H), 6.43 (dd, 1H, *J* = 8.4, 2.1 Hz, Ar-H), 5.64 (dd, *J* = 12.0, 5.1 Hz, 1H, pyrazoline-5H), 4.03 (dd, *J* = 17.7, 12.0 Hz, 1H, pyrazoline-4H), 3.86 (s, 3H, OCH_3), 3.81 (s, 3H, OCH_3), 3.23 (dd, *J* = 18.0, 5.1 Hz, 1H, pyrazoline-4H); ^{13}C NMR (DMSO-d_6, 75.5 MHz): δ 160.6, 157.8, 156.6, 152.8, 145.7, 133.2, 131.4, 128.7, 127.7, 120.6, 120.2, 116.8, 116.6, 112.1, 105.5, 99.4, 56.8 (C5-pyrazoline), 56.2 (OCH_3), 55.6 (OCH_3), 43.1 (C4-pyrazoline); DART MS: m/z 452.24 $[M+H]^+$, $C_{23}H_{23}N_3O_5SH^+$ Calcd. 452.510.

4-[5-(3,4-dimethoxyphenyl)-3-(2-hydroxyphenyl)-4,5-dihydro-1H-pyrazol-1-yl]benzenesulfonamide (7c)

M.p.198-200 °C (Lit. [10] 200 °C), yield 78%; IR (KBr, cm^{-1}): 3325, 3256 (m, N-H stretch), 1589 (s, C=N stretch), 1512 (s, N-H bend), 1327 and 1149 (s, SO_2 stretch); ^1H NMR (DMSO-d_6, 300 MHz): δ 10.36 (br s, ex, 1H, OH), 7.62 (d, 2H, *J* = 9.0 Hz, Ar-H), 7.51 (d, 1H, *J* = 6.6 Hz, Ar-H), 7.33-7.28 (m, 1H, Ar-H), 7.04 (s, ex, 2H, SO_2NH_2), 7.02-7.00 (m, 4H, Ar-H), 6.96 (d, 1H, *J* = 1.8 Hz, Ar-H), 6.88 (d, 1H, *J* = 8.4 Hz, Ar-H), 6.72 (dd, 1H, *J* = 8.4, 1.8 Hz, Ar-H), 5.52 (dd, *J* = 114, 5.1 Hz, 1H, pyrazoline-5H), 4.04 (dd, *J* = 17.7, 5.7 Hz, 1H, pyrazoline-4H), 3.72 (s, 3H,

OCH_3), 3.69 (s, 3H, OCH_3), 3.35 (merged with HOD peak of DMSO-d_6, 1H, pyrazoline-4H);^{13}C NMR (DMSO-d_6, 75.5 MHz): δ 160.6, 157.8, 156.6, 152.8, 145.7, 133.2, 131.4, 128.7, 127.7, 120.6, 120.2, 116.8, 116.6, 112.1, 105.5, 99.4, 56.8 (C5-pyrazoline), 56.2 (OCH_3), 55.6 (OCH_3), 43.1 (C4-pyrazoline); DART MS: m/z 452.45 $[M+H]^+$, $C_{23}H_{23}N_3O_5SH^+$ Calcd. 452.510.

4-[5-(2,3-dimethoxyphenyl)-3-(2-hydroxy-6-methoxyphenyl)-4,5-dihydro-1H-pyrazol-1yl] benzenesulfonamide (7d)

M.p. 222-224 °C, yield 83%; IR (KBr, cm^{-1}): 3742 (m, N-H stretch), 1589 (s, C=N stretch), 1512 (s, N-H bend), 1327 and 1149 (s, SO_2 stretch); ^{1}H NMR (DMSO-d_6, 300 MHz): δ 7.63 (d, 2H, *J* = 8.1 Hz, Ar-H), 7.24-7.21 (m, 1H, Ar-H), 7.04 (s, ex, 2H, SO_2NH_2), 6.98 (s, 3H, Ar-H), 6.91 (d, 2H, *J* = 8.4 Hz, Ar-H), 6.67 (br s, ex, 1H, OH), 6.61 (dd, 1H, *J* = 8.4 Hz), 5.61 (dd, *J* = 12.3, 5.4 Hz, 1H, pyrazoline-H), 4.11 (dd, *J* = 18.6, 12.3 Hz, 1H, pyrazoline-H), 3.8 (s, 6H, 2x OCH_3), 3.7 (s, 3H, OCH_3), 3.67 (merged with HOD peak of DMSO-d_6, 1H, pyrazoline-H); ^{13}C NMR (DMSO-d_6, 75.5 MHz): δ 159.1, 158.4, 153.1, 151.8, 146.1, 145.6, 134.8, 133.7, 131.6, 127.8, 124.9, 118.6, 112.9, 111.9, 109.7, 106.6, 103.1, 60.6 (C5-pyrazoline), 56.9 (OCH_3), 56.3 (OCH_3), 56.1 (OCH_3), 47.0 (C4-pyrazoline); DART MS: m/z 484. 50 $[M+H]^+$, $C_{24}H_{24}N_3O_6SH^+$ Calcd. 484.523.

4-[5-(2,4-dimethoxyphenyl)-3-(2-hydroxy-6-methoxyphenyl)-4,5-dihydro-1H-pyrazol-1yl] benzenesulfonamide (7e)

M.p. 198-200 °C, yield 81%; IR (KBr, cm^{-1}): 3742 (m, N-H stretch), 1589 (s, C=N stretch), 1504 (s, N-H bend), 1311 and 1149 (s, SO_2 stretch); ^{1}H NMR (DMSO-d_6, 300 MHz): δ 11.20 (br s, ex, 1H, OH), 7.61 (d, 2H, *J* = 8.4 Hz, Ar-H), 7.26.7.21 (m, 1H, Ar-H), 7.02 (s, ex, 2H, SO_2NH_2), 6.89-6.86 (m, 3H, Ar-H), 6.64 (d, 2H, *J* = 9.0 Hz, Ar-H), 6.59 -6.55 (m, 1H, Ar-H), 6.44 (dd, 1H, *J* = 8.4, 2.4 Hz, Ar-H), 5.58 (dd, *J* = 12.0, 4.8, Hz, 1H, pyrazoline-H), 4.06 (dd, *J* = 18.3, 12.3 Hz, 1H, pyrazoline-H), 3.85 (s, 3H, OCH_3), 3.76 (s, 3H, OCH_3), 3.71 (s, 3H, OCH_3), 3.24 (dd, *J* = 18.6, 5.4 Hz, 1H, pyrazoline-H); ^{13}C NMR (DMSO-d_6, 75.5 MHz): δ 160.6, 159.1, 158.3, 157.7, 151.8, 145.6, 133.4, 131.6, 127.7, 121.0, 111.9, 109.6, 106.7, 105.7, 103.1, 99.4, 56.3, 56.2, 55.6, 46.5 (C4-pyrazoline); DART MS: m/z 484.49 $[M+H]^+$, $C_{24}H_{24}N_3O_6SH^+$ Calcd. 484.523.

4-[5-(3,4-dimethoxyphenyl)-3-(2-hydroxy-6-methoxyphenyl)-4,5-dihydro-1H-pyrazol-1yl] benzenesulfonamide (7f)

M.p. 214-218 ℃, yield 84%; IR (KBr, cm^{-1}): 3742 (m, N-H stretch), 1589 (s, C=N stretch), 1512 (s, N-H bend), 1319 and 1157 (s, SO_2 stretch); ^1H NMR (DMSO-d_6, 300 MHz): δ 11.2 (br s, ex, 1H, OH), 7.62 (d, 2H, *J* = 8.7 Hz, Ar-H), 7.26.7.23 (m, 1H, Ar-H), 7.05 (s, ex, 2H, SO_2NH_2), 6.98 (d, 2H, *J* = 8.7 Hz, Ar-H), 6.89 (d, 2H, *J* = 8.4 Hz, Ar-H), 6.75 (dd, 1H, *J* = 8.4, 1.5 Hz, Ar-H), 6.61 (d, 2H, *J* = 8.1 Hz, Ar-H), 6.56 (d, 2H, *J* = 8.4 Hz, Ar-H), 5.44 (dd, *J* = 11.7, 5.4 Hz, 1H, pyrazoline-5H), 3.99 (dd, *J* = 16.8, 8.4 Hz, 1H, pyrazoline-4H), 3.75 (s, 3H, OCH_3), 3.73 (s, 3H, OCH_3), 3.70 (s, 3H, OCH_3), 3.37 (merged with HOD peak of DMSO-d_6, 1H, pyrazoline-4H); ^{13}C NMR (DMSO-d_6, 75.5 MHz): δ 159.1, 158.3, 151.2, 149.6, 148.7, 146.0, 134.3, 133.8, 131.5, 127.7, 118.2, 112.6, 112.3, 110.3, 109.6, 106.9, 103.0, 61.4 (C-5 pyrazoline), 56.3 (OCH_3), 56.0 (OCH_3), 55.9 (OCH_3), 47.8 (C4-pyrazoline); DART MS: m/z 484.48 $[M+H]^+$, $C_{24}H_{24}N_3O_6SH^+$ Calcd. 484.523.

4-[5-(2,3-dimethoxyphenyl)-3-(2-hydroxy-5-methylphenyl)-4,5-dihydro-1H-pyrazol-1-yl) benezenesulfonamide (7g)

M.p. 188-190 ℃, yield 80%; IR (KBr, cm^{-1}): 3279 (m, N-H stretch), 1589 (s, C=N stretch), 1481 (s, N-H bend), 1335 and 1149 (s, SO_2 stretch); ^1H NMR (DMSO-d_6, 300 MHz): δ 10.13 (br s, ex, 1H, OH), 7.62 (d, 2H, *J* = 8.7 Hz, Ar-H), 7.32 (s, 1H, Ar-H), 7.11 (d, *J* = 8.1 Hz, Ar-H), 7.04 (s, ex, 2H, SO_2NH_2), 6.98 (m, 3H, Ar-H), 6.81 (d, 1H, *J* = 8.4 Hz, Ar-H), 6.63-6.60 (m, 1H, Ar-H), 5.70 (dd, *J* =12.3, 5.1 Hz, 1H, pyrazoline-5H), 4.09 (dd, *J* = 17.7, 12.3 Hz, 1H, pyrazoline-4H), 3.82 (s, 3H, OCH_3), 3.81 (s, 3H, OCH_3), 3.36 (merged with HOD peak of DMSO-d_6, 1H, pyrazoline-4H), 2.24 (s, 3H, CH_3); ^{13}C NMR (DMSO-d_6, 75.5 MHz): δ 154.5, 153.2, 152.6, 146.2, 145.6, 134.6, 133.6, 132.0, 128.8, 128.7, 127.7, 124.9, 118.5, 116.5, 113.0, 112.1, 60.7 (C-5 pyrazoline), 57.3 (OCH_3), 56.1 (OCH_3), 43.8 (C4-pyrazoline), 20.4 (CH_3); DART MS: m/z 468.45 $[M+H]^+$, $C_{24}H_{24}N_3O_5SH^+$ Calcd. 468.529.

4-[5-(2,4-dimethoxyphenyl)-3-(2-hydroxy-5-methylphenyl)-4,5-dihydro-1H-pyrazol-1-yl) benezenesulfonamide (7h)

M.p. 228-230 °C, yield 82%; IR (KBr, cm^{-1}): 3364, 3256 (m, N-H stretch), 1589 (s, C=N stretch), 1497 (s, N-H bend), 1335 and 1149 (s, SO_2 stretch); ^{1}H NMR (DMSO-d_6, 300 MHz): δ 10.13 (br s, ex, 1H, OH), 7.61 (d, 2H, *J* = 8.7 Hz, Ar-H), 7.30 (s, 1H, Ar-H), 7.10 (d, *J* = 5.1 Hz, Ar-H), 7.03 (s, ex, 2H, SO_2NH_2), 6.93 (d, 2H, *J* = 8.7 Hz, Ar-H), 6.87 (d, 1H, *J* = 8.1 Hz, Ar-H), 6.81 (d, 1H, *J* = 8.4 Hz, Ar-H), 6.64 (d, 1H, *J* = 2.1 Hz, Ar-H), 6.42 (dd, 1H, *J* = 8.1, 2.1 Hz, Ar-H), 5.63 (dd, *J* =12.3, 4.8 Hz, 1H, pyrazoline-5H), 4.09 (dd, *J* = 18.0, 12.0 Hz, 1H, pyrazoline-H), 3.87 (s, 3H, OCH_3), 3.71 (s, 3H, OCH_3), 3.22 (dd, *J* = 17.7, 4.8 Hz, 1H, pyrazoline-H), 2.24 (s, 3H, CH_3); ^{13}C NMR (DMSO-d_6, 75.5 MHz): δ 160.6, 157.7, 154.5 152.8, 145.6, 133.5, 131.9, 128.8, 128.7, 127.7, 127.3, 120.6, 116.6, 116.5, 112.1, 105.5, 99.5, 56.7, 56.2, 55.6, 43.3 (C-4 pyrazoline), 20.4 (CH_3); DART MS: m/z 468.46 $[M+H]^+$, $C_{24}H_{24}N_3O_5SH^+$ Calcd. 468.529.

4-[5-(3,4-dimethoxyphenyl)-3-(2-hydroxy-5-methylphenyl)-4,5-dihydro-1H-pyrazol-1-yl) benezenesulfonamide (7i)

M.p. 194-198 °C, yield 77%; IR (KBr, cm^{-1}): 3365, 3256 (m, N-H stretch), 1589 (s, C=N stretch), 1497 (s, N-H bend), 1335 and 1149 (s, SO_2 stretch); ^{1}H NMR (DMSO-d_6, 300 MHz): δ^{1}H NMR (DMSO-d_6, 300 MHz): δ 10.11 (br s, ex, 1H, OH), 7.62 (d, 2H, *J* = 8.7 Hz, Ar-H), 7.33 (d, 1H, *J* = 5.7 Hz, Ar-H), 7.17 (d, *J* = 8.7 Hz, Ar-H), 7.04 (s, ex, 2H, SO_2NH_2), 7.01-6.98 (m, 2H, Ar-H), 6.96 (d, 1H, *J* = 1.5 Hz, Ar-H), 6.88 (d, 1H, *J* = 8.4 Hz, Ar-H), 6.70 (dd, 1H, *J* = 8.4, 1.8 Hz, Ar-H), 5.51 (dd, *J* =11.7, 5.4 Hz, 1H, pyrazoline-5H), 3.96 (dd, *J* = 16.8, 7.5 Hz, 1H, pyrazoline-4H), 3.73 (s, 3H, OCH_3), 3.70 (s, 3H, OCH_3), 3.47 (dd, *J* = 14.1, 6.9 Hz, 1H, pyrazoline-4H), 2.24 (s, 3H, CH_3); ^{13}C NMR (DMSO-d_6, 75.5 MHz): δ 154.5, 152.4, 149.5, 148.6, 146.0, 134.0, 133.6, 132.1, 128.8, 127.6, 117.9, 116.5, 112.6, 112.5, 110.2, 61.7, 56.6, 55.9, 55.9 (C-5 pyrazoline), 44.6 (C-4 pyrazoline), 20.3 (CH_3); DART MS: m/z 468.46 $[M+H]^+$, $C_{24}H_{24}N_3O_5SH^+$ Calcd. 468.529.

4-[3-(5-chloro-2-hydroxyphenyl)-5-(2,3-dimethoxyphenyl)-4,5-dihydro-1H-pyrazol-1yl) benzenesulfonamide (7j)

M.p. 180-182 °C, yield 78%; IR (KBr, cm^{-1}): 3362, 3255 (m, N-H stretch), 1589 (s, C=N stretch), 1497 (s, N-H bend), 1335 and 1149 (s, SO_2 stretch);^{1}H NMR (DMSO-d_6, 300 MHz): δ 10.43 (br s, ex, 1H, OH), 7.61 (d, 2H, *J* = 8.7 Hz, Ar-H), 7.58 (d, *J* = 2.4 Hz, Ar-H), 7.30 (dd, 1H, *J* = 8.7, 2.1 Hz, Ar-H), 7.04 (s, ex, 2H, SO_2NH_2), 7.00-6.97 (m, 3H, Ar-H), 6.84 (d, 1H, *J* = 8.4 Hz, Ar-

H), 6.63 (d, 1H, *J* = 1.8 Hz, Ar-H), 6.42 (dd, 1H, *J* = 8.1, 1.8 Hz, Ar-H), 5.64 (dd, *J* =11.7, 4.8 Hz, 1H, pyrazoline-5H), 4.00 (dd, *J* = 18.0, 12.3 Hz, 1H, pyrazoline-4H), 3.85 (s, 3H, OCH_3), 3.71 (s, 3H, OCH_3), 3.24 (dd, *J* =18.0, 5.1 Hz, 1H, pyrazoline-4H); ^{13}C NMR (DMSO-d_6, 75.5 MHz): δ 153.8, 153.0, 147.3, 146.8, 139.4, 133.3, 129.8, 124.9, 123.8, 122.9, 122.4, 120.1, 114.6, 61.1 (OCH_3), 55.9 (C-5 pyrazoline), 40.8 (C-4 pyrazoline); DART MS: m/z 488.89 $[M+H]^+$, $C_{24}H_{21}CN_3O_5SH^+$ Calcd. 488.947.

4-[3-(5-chloro-2-hydroxyphenyl)-5-(2,4-dimethoxyphenyl)-4,5-dihydro-1H-pyrazol-1yl) benzenesulfonamide (7k)

M.p. 218-220 °C, yield 75%; IR (KBr, cm^{-1}): 3365 and 3154 (m, N-H stretch),1589 (s, C=N stretch), 1497 (s, N-H bend),1335 and 1149 (s, SO_2 stretch); ^1H NMR (DMSO-d_6, 300 MHz): δ 10.43 (br s, ex, 1H, OH), 7.61 (d, 2H, *J* = 8.7 Hz, Ar-H), 7.58 (d, *J* = 2.4 Hz, Ar-H), 7.30 (dd, 1H, *J* = 8.7, 2.1 Hz, Ar-H), 7.04 (s, ex, 2H, SO_2NH_2), 7.00-6.97 (m, 3H, Ar-H), 6.84 (d, 1H, *J* = 8.4 Hz, Ar-H), 6.63 (d, 1H, *J* = 1.8 Hz, Ar-H), 6.42 (dd, 1H, *J* = 8.1, 1.8 Hz, Ar-H), 5.64 (dd, *J* =11.7, 4.8 Hz, 1H, pyrazoline-5H), 4.00 (dd, *J* = 18.0, 12.3 Hz, 1H, pyrazoline-H), 3.85 (s, 3H, OCH_3), 3.71 (s, 3H, OCH_3), 3.24 (dd, *J* =18.0, 5.1 Hz, 1H, pyrazoline-4H); ^{13}C NMR (DMSO-d_6, 75.5 MHz): δ 160.6, 157.7, 155.3, 151.0, 145.6, 133.8, 130.5, 127.7, 127.6, 123.7, 120.6, 119.1, 118.5, 112.3, 105.5, 99.5, 57.2 (C-5 pyrazoline), 56.2 (OCH_3), 55.6 (OCH_3), 43.4 (C-4 pyrazoline); DART MS: m/z 488.90 $[M+H]^+$, $C_{24}H_{21}CN_3O_5SH^+$ Calcd. 488.947.

4-[3-(5-chloro-2-hydroxyphenyl)-5-(3,4-dimethoxyphenyl)-4,5-dihydro-1H-pyrazol-1yl) benzenesulfonamide (7l)

M.p. 222-226 °C, yield 72%; IR (KBr, cm^{-1}): 3363, 3256 (m, N-H stretch), 1589 (s, C=N stretch), 1497 (s, N-H bend), 1335 and 1149 (s, SO_2 stretch); ^1H NMR (DMSO-d_6, 300 MHz): δ 10.40 (br s, ex, 1H, OH), 7.63-7.60 (m, 3H, Ar-H), 7.31 (dd, 1H, *J* = 9.0, 2.7 Hz, Ar-H), 7.07 (s, 2H, Ar-H), 7.04 (s, ex, 2H, SO_2NH_2), 6.99 (d, 1H, *J* = 8.7 Hz, Ar-H), 6.85 (d, 1H, *J* = 1.8 Hz, Ar-H), 6.88 (d, 1H, *J* = 8.4 Hz, Ar-H), 6.70 (dd, 1H, *J* = 8.4, 1.8 Hz, Ar-H), 5.53 (dd, *J* =12.3, 5.4 Hz, 1H, pyrazoline-5H), 4.06 (dd, *J* = 18.0, 12.0 Hz, 1H, pyrazoline-4H), 3.72 (s, 3H, OCH_3), 3.70 (s, 3H, OCH_3), 3.34 (merged with HOD peak of DMSO-d_6, 1H, pyrazoline-4H); ^{13}C NMR (DMSO-d_6, 75.5 MHz): δ 160.6, 157.7, 155.3, 151.0, 145.6, 133.8, 130.5, 127.7, 127.6, 123.7, 120.6,

119.1, 118.5, 112.3, 105.5, 99.5, 57.2 (C-5 pyrazoline), 56.2 (OCH_3), 55.6 (OCH_3), 43.4 (C-4 pyrazoline); DART MS: m/z 488.80 $[M+H]^+$, $C_{24}H_{21}CN_3O_5SH^+$ Calcd. 488.947.

4-[3-(2,6-dihydroxyphenyl)-5-(2,3-dimethoxyphenyl)-4,5-dihydro-1H-pyrazol-1-yl] benzenesulfonamide (7m)

M.p. 168-170 °C, yield 75%; IR (KBr, cm^{-1}): 3328, 3246 (m, N-H stretch), 1589 (s, C=N stretch), 1514 (s, N-H bend), 1327 and 1149 (s, SO_2 stretch);^{1}H NMR (DMSO-d_6, 300 MHz): δ 10.33 (br s, ex, 1H, OH), 7.62 (d, 2H, J = 8.7 Hz, Ar-H), 7.49 (d, 1H, J = 7.8 Hz, Ar-H), 7.32-7.27 (m, 1H, Ar-H), 7.04 (s, ex, 2H, SO_2NH_2), 7.00-6.89 (m, 4H, Ar-H), 6.64 (d, 1H, J = 2.4 Hz, Ar-H), 6.43 (dd, 1H, J = 8.4, 2.1 Hz, Ar-H), 5.64 (dd, J = 12.0, 5.1 Hz, 1H, pyrazoline-5H), 4.03 (dd, J = 17.7, 12.0 Hz, 1H, pyrazoline-4H), 3.86 (s, 3H, OCH_3), 3.81 (s, 3H, OCH_3), 3.23 (dd, J = 18.0, 5.1 Hz, 1H, pyrazoline-4H); ^{13}C NMR (DMSO-d_6, 75.5 MHz): δ 160.6, 157.8, 156.6, 152.8, 145.7, 133.2, 131.4, 128.7, 127.7, 120.6, 120.2, 116.8, 116.6, 112.1, 105.5, 99.4, 56.8 (C5-pyrazoline), 56.2 (OCH_3), 55.6 (OCH_3), 43.1 (C4-pyrazoline). DART MS: m/z 470.11 $[M+H]^+$, $C_{23}H_{23}N_3O_6SH^+$ Calcd. 470.110.

4-[3-(2,6-dihydroxyphenyl)-5-(2,4-dimethoxyphenyl)-4,5-dihydro-1H-pyrazol-1-yl] benzenesulfonamide (7n)

M.p. 162-164 °C, yield 78%; IR (KBr, cm^{-1}): 3328, 3246 (m, N-H stretch), 1589 (s, C=N stretch), 1514 (s, N-H bend), 1327 and 1149 (s, SO_2 stretch); ^{1}H NMR (DMSO-d_6, 300 MHz): δ 10.36 (br s, ex, 1H, OH), 7.62 (d, 2H, J = 8.7 Hz, Ar-H), 7.49 (d, 1H, J = 7.8 Hz, Ar-H), 7.32-7.27 (m, 1H, Ar-H), 7.04 (s, ex, 2H, SO_2NH_2), 7.00-6.89 (m, 4H, Ar-H), 6.64 (d, 1H, J = 2.4 Hz, Ar-H), 6.43 (dd, 1H, J = 8.4, 2.1 Hz, Ar-H), 5.64 (dd, J = 12.0, 5.1 Hz, 1H, pyrazoline-5H), 4.03 (dd, J = 17.7, 12.0 Hz, 1H, pyrazoline-4H), 3.86 (s, 3H, OCH_3), 3.81 (s, 3H, OCH_3), 3.23 (dd, J = 18.0, 5.1 Hz, 1H, pyrazoline-4H); ^{13}C NMR (DMSO-d_6, 75.5 MHz): δ 159.6, 157.8, 155.6, 152.8, 145.7, 133.2, 130.4, 128.7, 127.7, 120.6, 120.1, 116.8, 115.6, 112.1, 105.5, 99.4, 56.8 (C5-pyrazoline), 56.2 (OCH_3), 55.6 (OCH_3), 43.1 (C4-pyrazoline); DART MS: m/z 470.18 $[M+H]^+$, $C_{23}H_{23}N_3O_6SH^+$ Calcd. 470.110.

4-[3-(2,6-dihydroxyphenyl)-5-(3,4-dimethoxyphenyl)-4,5-dihydro-1H-pyrazol-1-yl] benzenesulfonamide (7o)

M.p. 198-200 °C, yield 71%; IR (KBr, cm^{-1}): 3328, 3246 (m, N-H stretch), 1589 (s, C=N stretch), 1514 (s, N-H bend), 1327 and 1149 (s, SO_2 stretch);^{1}H NMR (DMSO-d_6, 300 MHz): δ 10.36 (br s, ex, 1H, OH),7.62 (d, 2H, *J* = 8.7 Hz, Ar-H), 7.49 (d, 1H, *J* = 7.8 Hz, Ar-H), 7.32-7.27 (m, 1H, Ar-H), 7.04 (s, ex, 2H, SO_2NH_2), 7.00-6.89 (m, 4H, Ar-H), 6.64 (d, 1H, *J* = 2.4 Hz, Ar-H), 6.43 (dd, 1H, *J* = 8.4, 2.1 Hz, Ar-H), 5.64 (dd, *J* = 12.0, 5.1 Hz, 1H, pyrazoline-5H), 4.03 (dd, *J* = 17.7, 12.0 Hz, 1H, pyrazoline-4H), 3.86 (s, 3H, OCH_3), 3.81 (s, 3H, OCH_3), 3.23 (dd, *J* = 18.0, 5.1 Hz, 1H, pyrazoline-4H); ^{13}C NMR (DMSO-d_6, 75.5 MHz): δ 160.7, 157.8, 156.6, 152.8, 145.7, 133.2, 131.4, 128.7, 127.7, 120.6, 120.2, 116.8, 116.6, 112.1, 105.5, 99.4, 56.8 (C5-pyrazoline), 56.2 (OCH_3), 55.6 (OCH_3), 43.1 (C4-pyrazoline); DART MS: m/z 470.19 $[M+H]^+$, $C_{23}H_{23}N_3O_6SH^+$ Calcd. 470.110.

4-[3-(2,4-dihydroxyphenyl)-5-(2,3-dimethoxyphenyl)-4,5-dihydro-1H-pyrazol-1-yl] benzenesulfonamide (7p)

M.p. 170-174 °C, yield 76%; IR (KBr, cm^{-1}): 3310, 3263 (m, N-H stretch), 1597 (s, C=N stretch), 1504 (s, N-H bend), 1327 and 1149 (s, SO_2 stretch); ^{1}H NMR (DMSO-d_6, 300 MHz): δ 10.36 (br s, ex, 1H, OH), 7.61 (d, 2H, *J* = 8.7 Hz, Ar-H), 7.50 (d, 1H, *J* = 7.8 Hz, Ar-H), 7.32-7.26 (m, 1H, Ar-H), 7.04 (s, ex, 2H, SO_2NH_2), 7.00-6.90 (m, 4H, Ar-H), 6.64 (d, 1H, *J* = 2.4 Hz, Ar-H), 6.42 (dd, 1H, *J* = 8.4, 2.1 Hz, Ar-H), 5.55 (dd, *J* = 12.0, 5.1 Hz, 1H, pyrazoline-5H), 4.03 (dd, *J* = 17.7, 12.0 Hz, 1H, pyrazoline-4H), 3.87 (s, 3H, OCH_3), 3.80 (s, 3H, OCH_3), 3.23 (dd, *J* = 18.0, 5.1 Hz, 1H, pyrazoline-4H); ^{13}C NMR (DMSO-d_6, 75.5 MHz): δ 160.6, 157.8, 156.6, 152.8, 145.7, 133.2, 131.4, 128.7, 127.7, 120.6, 120.2, 116.8, 116.6, 112.1, 105.5, 99.4, 56.8 (C5-pyrazoline), 56.2 (OCH_3), 55.6 (OCH_3), 43.1 (C4-pyrazoline); ^{13}C NMR (DMSO-d_6, 75.5 MHz): δ 157.2, 157.3, 148.4, 149.2, 147.5, 146.3, 134.9, 132.4, 131.1, 128.0, 119.5, 112.1, 111.9, 110.9, 109.4, 108.1, 106.3, 56.0 (C5-pyrazoline), 56.2 (OCH_3), 55.6 (OCH_3), 40.5 (C4-pyrazoline); DART MS: m/z 470.12 $[M+H]^+$, $C_{23}H_{23}N_3O_6SH^+$ Calcd. 470.110.

4-[3-(2,4-dihydroxyphenyl)-5-(2,4-dimethoxyphenyl)-4,5-dihydro-1H-pyrazol-1-yl] benzenesulfonamide (7q)

M.p. 210-212 °C, yield 69%; IR (KBr, cm^{-1}): 3387, 3279 (m, N-H stretch), 1597 (s, C=N stretch), 1504 (s, N-H bend), and 1335 and 1157 (s, SO_2 stretch); ^{1}H NMR (DMSO-d_6, 300 MHz): δ 12.05 (br s, ex, 1H, OH), 10.08 (br s, ex, 1H, OH), 7.71 (d, 2H, J = 8.7 Hz, Ar-H), 7.44 (d, 1H, J = 7.8 Hz, Ar-H), 7.14 (s, ex, 2H, SO_2NH_2), 7.10 (d, 1H, J = 8.7 Hz), 6.62-6.50 (m, 4H, Ar-H), 6.64 (d, 1H, J = 2.4 Hz, Ar-H), 6.43 (dd, 1H, J = 8.4, 2.1 Hz, Ar-H), 5.64 (dd, J = 12.0, 5.1 Hz, 1H, pyrazoline-5H), 4.03 (dd, J = 17.7, 12.0 Hz, 1H, pyrazoline-4H), 3.86 (s, 3H, OCH_3), 3.81 (s, 3H, OCH_3), 3.14 (dd, J = 18.0, 5.1 Hz, 1H, pyrazoline-4H); ^{13}C NMR (DMSO-d_6, 75.5 MHz): δ 160.6, 157.8, 156.6, 152.8, 145.7, 133.2, 131.4, 128.7, 127.7, 120.6, 120.2, 116.8, 116.6, 112.1, 105.5, 99.4, 56.8 (C5-pyrazoline), 56.2 (OCH_3), 55.6 (OCH_3), 43.1 (C4-pyrazoline); DART MS: m/z 470.1 $[M+H]^+$, $C_{23}H_{23}N_3O_6SH^+$ Calcd. 470.110.

4-[3-(2,4-dihydroxyphenyl)-5-(3,4-dimethoxyphenyl)-4,5-dihydro-1H-pyrazol-1-yl] benzenesulfonamide (7r)

M.p. 170-172 °C, yield 76%; IR (KBr, cm^{-1}): 3379, 3310 (m, N-H stretch), 1589 (s, C=N stretch), 1509 (s, N-H bend), and 1335 and 1149 (s, SO_2 stretch); ^{1}H NMR (DMSO-d_6, 300 MHz): δ 10.05 (br s, ex, 1H, OH), 7.72 (d, 2H, J = 8.4 Hz, Ar-H), 7.49 (d, 1H, J = 7.8 Hz, Ar-H), 7.32-7.27 (m, 1H, Ar-H), 7.04 (s, ex, 2H, SO_2NH_2), 7.00-6.89 (m, 4H, Ar-H), 6.64 (d, 1H, J = 2.4 Hz, Ar-H), 6.43 (dd, 1H, J = 8.4, 2.1 Hz, Ar-H), 5.77 (dd, J = 12.0, 5.1 Hz, 1H, pyrazoline-5H), 4.03 (dd, J = 17.7, 12.0 Hz, 1H, pyrazoline-4H), 3.78 (s, 3H, OCH_3), 3.77 (s, 3H, OCH_3), 3.11 (dd, J = 18.0, 5.1 Hz, 1H, pyrazoline-4H); ^{13}C NMR (DMSO-d_6, 75.5 MHz): δ 160.6, 157.8, 156.6, 152.8, 145.7, 133.2, 131.4, 128.7, 127.7, 120.6, 120.2, 116.8, 116.6, 112.1, 105.5, 99.4, 56.8 (C5-pyrazoline), 56.2 (OCH_3), 55.6 (OCH_3), 43.1 (C4-pyrazoline); DART MS: m/z 470.15 $[M+H]^+$, $C_{23}H_{23}N_3O_6SH^+$ Calcd. 470.110.

References

1. Tran TD, Park H, Kim HP, Ecker GF, Thai KM (2009) Inhibitory activity of prostaglandin E2 production by the synthetic 2'-hydroxychalcone analogues: Synthesis and SAR study. Bioorg. Med. Chem. Lett. 19: 1650-1653.

2. Wu L, Li J, Cai Y, Pan Y, Ye F, et al. (2011) Evaluation and discovery of novel synthetic chalcone derivatives as anti-inflammatory agents. J. Med. Chem. 54: 8110-8123.

3. Sagrera G, Bertucci A, Vazquez A, Seoane G Bioorg (2011) Synthesis and antifungal activities of natural and synthetic biflavonoids. Med. Chem. 19: 3060-3073.

4. Mewett KN, Fernandez SP, Pasricha AK, Ponga A, Devenisha SO, et al. (2009) Synthesis and biological evaluation of flavan-3-ol derivatives as positive modulators of GABAA receptors. Bioorg. Med. Chem. 17: 7156-7173.

5. Turov AV, Bondarenko SP, Tkachuk AA, Khilya VP (2005) Conformational Mobility of Substituted 2-Methoxychalcones under the Action of Lanthanide Shift Reagents. Russian Journal of Organic Chemistry. 41(1): 47-53.

6. Miles CO, Main L, Nicholson BK (1989) Synthesis of 2', 6'-Dihydroxychalcones by Using Tetrahydropyran-2-yl and Trialkylsilyl Protective Groups; the Crystal Structure Determination of 2',6'-Dihydroxy-2,4,6-trimethoxychalcone. Australian J. Chem, 42(7): 1103-1113.

7. Dhar DN, Lal JB (1958) Chalcones. Condensation of Aromatic Aldehydes with Resacetophenone. II. J. Org. Chem. 23(8): 1159-1161.

8. Neves MP, Cravo S, Lima RT, Vasconcelos MH, Nascimento MS, et al. (2012) Solid-phase synthesis of 2'-hydroxychalcones. Effects on cell growth inhibition, cell cycle and apoptosis of human tumor cell lines. Bioorg. Med. Chem. 20: 25-33.

9. Juvale K, Pape VF, Wiese, M. (2012) Investigation of chalcones and benzochalcones as inhibitors of breast cancer resistance protein. Bioorg. Med. Chem. 20(1): 346-55.

10. Rathisha IG, Javeda K, Ahmada S, Bano S, Alama MS, et al. (2009) Synthesis and antiinflammatory activity of some new 1,3,5-trisubstituted pyrazolines bearing benzene sulfonamide. Bio. Org. Med. Chem. Lett. 19: 255-258.
